# Identified members of the *Streptomyces lividans* AdpA regulon involved in differentiation and secondary metabolism

**DOI:** 10.1186/1471-2180-14-81

**Published:** 2014-04-03

**Authors:** Aurélie Guyet, Nadia Benaroudj, Caroline Proux, Myriam Gominet, Jean-Yves Coppée, Philippe Mazodier

**Affiliations:** 1 Unité de Biologie des Bactéries Pathogènes à Gram-Positif, Institut Pasteur, CNRS URA 2172, 28 rue du Docteur Roux, 75724 Paris Cedex 15, France; 2 Unité Biologie des Spirochètes, Institut Pasteur, 28 rue du Docteur Roux, 75724 Paris Cedex 15, France; 3 Plateforme Transcriptome et Epigenome (PF2), 28 rue du Docteur Roux, 75724 Paris Cedex 15, France

**Keywords:** *Streptomyces*, *lividans*, Microarrays, AdpA, *bldA*, *ramR*, *hyaS*, CYP105D5, *cchB*, Coelichelin

## Abstract

**Background:**

AdpA is a key transcriptional regulator involved in the complex growth cycle of *Streptomyces. Streptomyces* are Gram-positive bacteria well-known for their production of secondary metabolites and antibiotics. Most work on AdpA has been in *S. griseus*, and little is known about the pathways it controls in other *Streptomyces* spp. We recently discovered interplay between ClpP peptidases and AdpA in *S. lividans*. Here, we report the identification of genes directly regulated by AdpA in *S. lividans*.

**Results:**

Microarray experiments revealed that the expression of hundreds of genes was affected in a *S. lividans adpA* mutant during early stationary phase cultures in YEME liquid medium. We studied the expression of the *S. lividans* AdpA-regulated genes by quantitative real-time PCR analysis after various times of growth. *In silico* analysis revealed the presence of potential AdpA-binding sites upstream from these genes; electrophoretic mobility shift assays indicated that AdpA binds directly to their promoter regions. This work identifies new pathways directly controlled by AdpA and that are involved in *S. lividans* development (*ramR*, SLI7885 also known as *hyaS* and SLI6586), and primary (SLI0755-SLI0754 encoding CYP105D5 and Fdx4) or secondary (*cchA*, *cchB,* and *hyaS*) metabolism.

**Conclusions:**

We characterised six *S. lividans* AdpA-dependent genes whose expression is directly activated by this pleiotropic regulator. Several of these genes are orthologous to *bldA*-dependent genes in *S. coelicolor*. Furthermore, *in silico* analysis suggests that over hundred genes may be directly activated or repressed by *S. lividans* AdpA, although few have been described as being part of any *Streptomyces* AdpA regulons. This study increases the number of known AdpA-regulated pathways in *Streptomyces* spp.

## Background

Streptomycetes are Gram-positive soil bacteria that display a complex morphological and metabolic differentiation. *Streptomyces* develop branched hyphae that expand by tip extension to form a vegetative mycelium meshwork. In response to as yet unidentified signals and to nutritient depletion, aerial branches emerge from the surface of colonies and may produce spores. As the aerial mycelium develops, *Streptomyces* colonies produce diverse secondary metabolites and synthesise antibiotics [1]. This differentiation cycle can be reproduced in laboratory conditions by growing *Streptomyces* cells on solid media. Most *Streptomyces* species do not form aerial mycelium or spores when in liquid media (e.g. *S. coelicolor* and *S. lividans*), and antibiotic production occurs in submerged cultures [2].

AdpA, also known as BldH, has been identified as a conserved major transcriptional regulator involved in the formation of aerial mycelia in various *Streptomyces* species [3-6]. AdpA is a member of the family of AraC/XylS regulator proteins that contain a C-terminal domain with two helix-turn-helix DNA-binding motifs; these features are strictly conserved in all *Streptomyces* AdpAs in the StrepDB database [7]. The N-terminal domain of AdpA is responsible for its dimerization and regulation [8,9]. Protein/DNA interaction experiments identified the following consensus AdpA-binding site in *S. griseus*: 5′-TGGCSNGWWY-3′ (with S: G or C; W: A or T; Y: T or C; N: any nucleotide) [10].

AdpA was discovered and has mostly been studied in *S. griseus*, in which it was first shown to activate expression of about thirty genes directly. They include genes encoding secreted proteins (e.g. proteases), a sigma factor (AdsA), a subtilisin inhibitor (SgiA)*,* SsgA which is essential for spore septum formation and the AmfR transcriptional regulator involved in production of AmfS (known as SapB in *S. coelicolor*), a small hydrophobic peptide involved in the emergence of aerial hyphae [11,12]. AdpA also plays a role in secondary metabolism and directly activates streptomycin biosynthesis [3].

Proteomic, transcriptomic and ChIP-sequencing analyses revealed that, in fact, several hundred genes are under the control of *S. griseus* AdpA and that AdpA acts as transcriptional activator as well as repressor [12-15]. In *S. coelicolor*, few genes have been identified as being directly regulated by AdpA: *sti1* (*sgiA* orthologs), *ramR* (*amfR* orthologs), *clpP1* (encoding a peptidase) [16] and *wblA* (encoding a transcriptional regulator) [15].

The regulation of *adpA* gene expression is complex and various mechanisms have been described [17]. AdpA represses its own gene expression in *S. griseus*[18] whereas it activates its own transcription in *S. coelicolor*[16]. In several *Streptomyces* species, the binding of γ-butyrolactones to a γ-butyrolactone receptor represses the *adpA* promoter [19,20]. In *S. coelicolor*, BldD represses *adpA* expression [21]. At the translational level, a feedback-control loop regulates levels of AdpA and AbsB (a RNAse III) in *S. coelicolor*[22,23]. A positive feedback loop between AdpA and BldA, the only tRNA able to read the UUA codon present in all *adpA* mRNA, has been demonstrated in *S. griseus*[22,23]. In *S. coelicolor, adpA* expression is constant during growth in liquid media [4] whereas on solid media, *adpA* is strongly expressed before aerial hyphae formation and AdpA is most abundant during the early aerial mycelium stage [4,16].

Even though AdpA plays a major role in development of *Streptomyces* spp., little is known about the pathways it controls in *S. lividans,* a species closely related to *S. coelicolor* and whose genome has recently been sequenced [24]. We have recently shown that in *S. lividans* AdpA directly controls *sti1* and the *clpP1clpP2* operon, encoding important factors for *Streptomyces* differentiation; we also found interplay between AdpA and ClpP1 [25]. Here, we report microarray experiments, quantitative real-time PCR (qRT-PCR), *in silico* analysis and protein/DNA interaction studies that identify other genes directly regulated by AdpA in *S. lividans*. Finally, *in silico* genome analysis allowed the identification of over hundred genes that are probably directly activated or repressed by AdpA in *S. lividans*. These findings and observations reveal new AdpA-dependent pathways in *S. lividans*.

## Methods

### Bacterial strains, growth conditions and media

*S. lividans* 1326 was obtained from the John Innes Culture Collection. In this *S. lividans* background, we constructed an *adpA* mutant in which *adpA* was replaced with an apramycin-resistance cassette [25].

*Streptomyces* was grown on NE plates [26] and in YEME liquid medium [27] in baffled flasks. MS medium was used for sporulation experiments [27]. Apramycin was added to final concentrations of 25 μg mL^-1^ to solid media and 20 μg mL^-1^ to liquid media as appropriate.

### Microarray experiments

**Figure 1:**
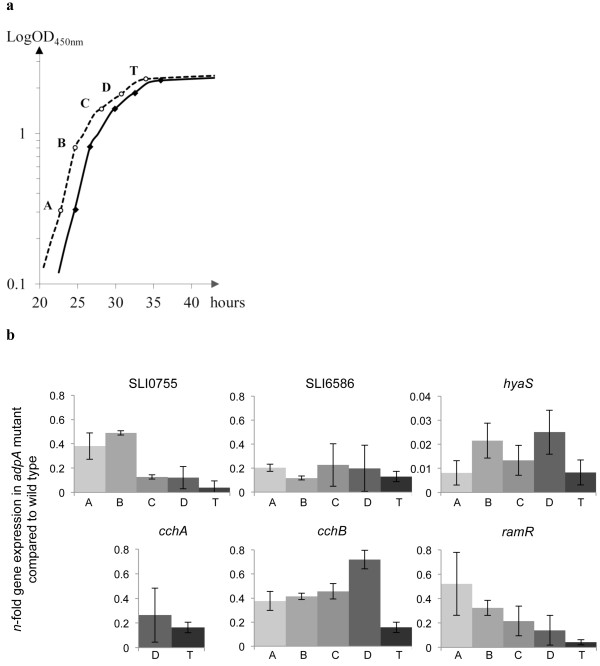
**Effects of *S. lividans adpA *mutation on expression of selected genes. a**. Growth curve of wild-type *S. lividans* (dashed line) and *adpA* mutant (solid line) in YEME liquid medium at 30°C with shaking at 200 rpm as followed by measuring absorbance at 450 nm. A, B, C, D and T indicate the time points when cultures were harvested for RNA extraction. Microarray experiments were performed on RNA samples extracted at time T. **b**. Change in gene expression *S. lividans adpA* mutant compared to the wild-type at each time point of growth. RNA was extracted from *S. lividans* wild-type 1326 and *adpA* mutant cells cultivated in liquid YEME medium after various times of growth (OD_450nm_ of 0.3, 0.8, 1.5, 1.9 and 2.3, respectively, at time points A, B, C, D and T). Relative amounts of SLI0755, SLI6586, *hyaS*, *cchA*, *cchB*, *ramR* PCR product were measured by qRT-PCR. At each time point of growth, gene expression levels were normalized using *hrdB* as an internal reference and are indicated in this figure as the *n*-fold change in *adpA* mutant compared to the wild type. Results are expressed as means and standard deviations of at least three replicates. Data are representative of at least two independent experiments for each strain at each growth time. Note that a different scale is used for *hyaS*.

*S. lividans* microarrays were not available, so *S. coelicolor* oligonucleotide arrays covering most open reading frames (ORFs) of the genome (for array coverage and design, see [28,29]) were used. Aliquots of 60 mL of liquid YEME medium were inoculated with about 10^8^ spores and incubated at 30°C with shaking at 200 rpm until early stationary phase (about 30 h of growth). Samples of 12 mL of culture (at OD_450nm_ = 2.3, corresponding to time point T on Figure 1a) were then collected and RNA extracted as previously described [30]. RNA quality was assessed with an Agilent 2100 Bioanalyser (Agilent Technologies). RNA indirect labelling and array hybridization were performed as described [31] and hybridized microarrays were scanned with a Genepix 4000A scanner (Molecular Devices).

### Statistical analysis of array data

R software [32] was used for normalization and differential analysis. A Loess normalization [33] was performed on a slide-by-slide basis (BioConductor package marray; [34]). A paired *t*-test was used for differential analysis. Variance estimates for each gene were computed under the hypothesis of homoscedasticity, together with the Benjamini and Yekutieli *P*-value adjustment method [35]. Only genes with a significant (*P*-value < 0.05) fold change (Fc) were taken into consideration. Empty and flagged spots were excluded, and only genes with no missing values were analysed. A few genes which displayed excessive variation were analysed using the Vmixt method from the VarMixt package [36]. We defined our cut-off for microarray data acquisition as Fc <0.625 or Fc > 1.6 with *P*-value < 0.05. The genome of *S. lividans* 1326 was sequenced only recently [24], so we used the StrepDB database [7], and in some cases a basic local alignment search tool (Blast), to identify *S. lividans* orthologs (SLI gene number) of *S. coelicolor* genes*.* We also used the protein classification scheme for the *S. coelicolor* genome available on the Welcome Trust Sanger Institute database [37].

### qRT-PCR analysis

Oligonucleotide pairs specific for *cchA* (SLI0459), *cchB* (SLI0458), SLI0755, SLI6586, *ramR* (SLI7029), *hyaS* (SLI7885) and *hrdB* (SLI6088, MG16-17) (Additional file 1: Table S1) were designed using the BEACON Designer software (Premier BioSoft). RNA samples were extracted from cultures in YEME liquid medium at OD_450nm_ values of about 0.3, 0.8, 1.5, 1.9 and 2.3 (time points A, B, C, D and T, respectively). Aliquots of 20 μg of RNA were treated twice with 2 Units of DNase I with the TURBO DNA-free reagent (Ambion) for 30 min at 37°C. Reverse transcription and quantitative real-time PCR were performed as previously described [25]. PCRs involved a hybridization step of 55°C, except for *ramR,* SLI0755 and *cchB* where a temperature of 58°C was used. Each assay was performed in triplicate and repeated with at least two independent RNA samples. The critical threshold cycle (*C*_*T*_) was defined for each sample. The relative amounts of cDNA for the tested genes were normalized to that of the *hrdB* gene transcript which did not vary under our experimental conditions (and thus served as an internal standard). The change (*n*-fold) in a transcript level was calculated using the following equations: *ΔC*_*T*_ = *C*_*T*(*test* DNA)_ - *C*_*T*(*reference* cDNA)_, *ΔΔC*_*T*_ = *ΔC*_*T*(target gene)_ - *ΔC*_*T*(hrdB)_, and ratio = $2^{-\Delta \Delta C_{T}}$[38]. Student’s *t* test was used to evaluate the significance of differences between the expression level of tested genes and that of a reference gene. A *P*-value < 0.05 was considered significant.

### *In silico* analysis and electrophoretic mobility shift assays (EMSA)

Several AdpA-binding site sequences, identified in *S. griseus* by DNase I footprinting experiments [10,13,18,23], were used with the PREDetector software (version 1.2.3.0) [39] to generate a *S. griseus* matrix [25]. This matrix was used with the *S. coelicolor* genome sequence (the *S. lividans* genome sequence was not available during the course of this study and is still not available on PREDetector software) to identify putative AdpA-binding sites upstream from *S. lividans* AdpA-dependent genes (scores > 3). The StrepDB database [7] and Blast were used to identify *S. lividans*, *S. coelicolor* and *S. griseus* ortholog gene names.

Radioactively labelled DNA fragments (180 bp to 496 bp) corresponding to promoter regions of putative *S. lividans* AdpA-regulated genes were obtained by PCR. Primers (named GSgene in Additional file 1: Table S1) were used to amplify the promoter regions of *cchA* (opposite orientation to *cchB*), SLI0755, SLI6586 (opposite orientation to SLI6587), *ramR* and *hyaS* as described elsewhere [25]. Purified radiolabelled fragments (10,000 cpm) were then used with purified AdpA histidine-tagged protein (AdpA-His_6_) in EMSA as previously described [25,40].

## Results

### Deletion of *adpA* affects the expression of hundreds of genes during early stationary phase

We had previously inactivated *adpA* in *S. lividans* and found that this *adpA* mutant failed to produce aerial mycelium on rich media and that its growth was comparable to that of the parental strain 1326 in liquid YEME medium at 30°C [25]. Expression studies with this *S. lividans adpA* mutant cultivated in liquid medium identified two differentiation-regulating factors (STI1 and the ClpP1ClpP2 peptidases) whose ORFs were under the direct control of AdpA [25]. We used transcriptome analysis of this *adpA* mutant to identify other AdpA-dependent pathways in *S. lividans*; however, this analysis was performed using *S. coelicolor* microarrays [29] because the *S. lividans* genome sequence was not yet available [24] and the two species are very closely related [41]. Total RNA was isolated from *S. lividans* 1326 and *adpA* cells during early stationary phase (time point T in Figure 1a) because at this growth phase, *S. coelicolor adpA* is expressed [4]; also the expression of genes involved in secondary metabolism in a *S. coelicolor bldA* mutant [42], a strain defective for AdpA translation, starts to diverge from that in the wild-type.

Global gene expression in the mutant was compared to that in the parental strain. The expression of more than 300 genes was affected in the *adpA* mutant at early stationary phase (Table 1 and Additional file 2: Table S2): 193 genes were significantly down-regulated (1.6-to 30-fold i.e. 0.033 < Fc < 0.625), and 138 were up-regulated (1.6-to 3.6-fold) with a *P*-value < 0.05 (see Additional file 2: Table S2 for the complete data set). Theses genes encode proteins of several different classes according to the Welcome Trust Sanger Institute *S. coelicolor* genome database [37]: 72 of the genes are involved in metabolism of small molecules, including seven playing a role in electron transport (e.g. SLI0755-SLI0754, *cydAB* operons) (Table 1); 18 encode proteins involved in secondary metabolism, for example the *cchA-cchF* gene cluster (SLI0459-0454) involved in coelichelin biosynthesis [43] and the SLI0339-0359 cluster encoding the putative deoxysugar synthase/glycosyltransferase. Deletion of *adpA* in *S. lividans* also affected the expression of 32 genes involved in regulation including *ramR* (SLI7029), *wblA* (SLI3822), *bldN* (SLI3667), *hrdD* (SLI3556) and *cutRS* (SLI6134-35) [1,6]. Sixty-two genes involved in the cell envelope [37] were differentially expressed in the *adpA* mutant; they include *hyaS* (SLI7885) [44], *chpE*, *chpH*[1], SLI6586 and SLI6587 which were strongly down-regulated in the *adpA* mutant (Table 1). Thirty-nine genes encoding proteins involved in various cellular processes (osmotic adaptation, transport/binding proteins, chaperones, and detoxification) [37] were also deregulated in the absence of AdpA (Additional file 2: Table S2). The expression of 111 genes coding for proteins with unidentified or unclassified function was altered in the *adpA* mutant. Thus, deletion of *adpA* influenced the expression of a large number of genes involved in a broad range of metabolic pathways, and indeed other functions, in *S. lividans*.

**Table 1:** **Genes differentially expressed in *S. lividans adpA *** **mutant at early stationary phase in YEME medium**^**a**^

***S. coelicolor *** **gene**^**b**^	***S. lividans *** **gene**^**c**^	**Other gene names**^**d**^	**Annotated function**^**b**^	**Fc**^**e**^	**Class or metabolism**^**f**^
SCO0382	SLI0340		UDP-glucose/GDP-mannose family dehydrogenase	0.491	Secondary (s. m.)
SCO0383	SLI0341		Hypothetical protein SCF62.09	0.527	Secondary (s. m.)
SCO0384	SLI0342		Putative membrane protein	0.611	Secondary (s. m.)
SCO0391	SLI0349		Putative transferase	0.613	Secondary (s. m.)
SCO0392	SLI0350		Putative methyltransferase	0.606	Secondary (s. m.)
SCO0394	SLI0352		Hypothetical protein SCF62.20	0.518	Secondary (s. m.)
SCO0396	SLI0354		Hypothetical protein SCF62.22	0.454	Secondary (s. m.)
SCO0397	SLI0355		Putative integral membrane protein	0.312	Secondary (s. m.)
SCO0399	SLI0357		Putative membrane protein	0.532	Secondary (s. m.)
SCO0494	SLI0454	*cchF*	Putative iron-siderophore binding lipoprotein	0.615	Secondary (s. m.)
SCO0496	SLI0456	*cchD*	Putative iron-siderophore permease transmembrane protein	0.505	Secondary (s. m.)
SCO0497	SLI0457	*cchC*	Putative iron-siderophore permease transmembrane protein	0.492	Secondary (s. m.)
SCO0498	SLI0458*	*cchB*	Putative peptide monooxygenase	0.336	Secondary (s. m.)
SCO0499	SLI0459*	*cchA*	Putative formyltransferase	0.374	Secondary (s. m.)
SCO0762	SLI0743	*sti1, sgiA*	Protease inhibitor precursor	0.124	(m. m.)
SCO0773	SLI0754	*soyB2*	Putative ferredoxin, Fdx4	0.098	Electron transport (s. m.)
SCO0774	SLI0755*		Putative cytochrome P450, CYP105D5	0.075	Electron transport (s. m.)
SCO0775	SLI0756*		Conserved hypothetical protein	0.424	Unknown function
SCO1630-28	SLI1934-32	*rarABC, cvnABC9*	Putative integral membrane protein	± 0.43	Cell envelope
SCO1674	SLI1979	*chpC*	Putative secreted protein	0.564	Cell envelope
SCO1675	SLI1980	*chpH*	Putative small membrane protein	0.237	Cell envelope
SCO1800	SLI2108	*chpE*	Putative small secreted protein	0.256	Cell envelope
SCO2780	SLI3127	*desE*	Putative secreted protein	1.757	Cell envelope
SCO2792	SLI3139	*bldH, adpA*	araC-family transcriptional regulator	0.383	Regulation
SCO2793	SLI3140	*ornA*	Oligoribonuclease	1.966	(m. m.)
SCO3202	SLI3556	*hrdD*	RNA polymerase principal sigma factor	2.499	Regulation
SCO3323	SLI3667	*bldN, adsA*	Putative RNA polymerase sigma factor	0.389	Regulation
SCO3579	SLI3822	*wblA*	Putative regulatory protein	0.310	Regulation
SCO3945	SLI4193	*cydA*	Putative cytochrome oxidase subunit I	3.386	Electron transport (s. m.)
SCO3946	SLI4194	*cydB*	Putative cytochrome oxidase subunit II	3.594	Electron transport (s. m.)
SCO4114	SLI4345		Sporulation associated protein	0.487	Cell envelope
SCO5240	SLI5531	*wblE*	Hypothetical protein	2.246	Unknown function
SCO5862-63	SLI6134-35	*cutRS*	Two-component regulator/sensor	± 1.82	Regulation
SCO6197	SLI6586*		Putative secreted protein	0.147	Cell envelope
SCO6198	SLI6587*		Putative secreted protein	0.618	Cell envelope
SCO6685	SLI7029*	*ramR, amfR*	Putative two-component system response regulator	0.624	Regulation
SCO7400-398	SLI7619-17	*cdtCBA*	Putative ABC-transport protein	± 1.75	Cell process
SCO7657	SLI7885*	*hyaS*	Putative secreted protein	0.033	Cell envelope
SCO7658	detected		Hypothetical protein SC10F4.31	0.103	Unknown function

^a^Gene expression in the *S. lividans adpA* mutant was compared to that in the wild-type, using *S. coelicolor* microarrays. Table 1 shows a selected subset of the genes (see Additional file 2: Table S2 for the complete list). The genes presented here were further studied or are discussed in the text because of their role in *Streptomyces* primary or secondary metabolism [1,6,17].

^b^Gene names for *S. coelicolor* (SCO) and *S. lividans* (SLI) and annotated function are from the StrepDB database [7].

^c^*S. coelicolor* microarrays were used for transcriptome analysis of the *S. lividans adpA* mutant (the complete microarray data set is presented in Additional file 2: Table S2). The *S. lividans* genome sequence was recently made available [24] and SLI ortholog gene numbers were identified as SCO gene orthologs with StrepDB database [7]. The expression of genes shown in bold was analysed by qRT-PCR. Intergenic DNA regions between genes labelled with asterisks were analyzed by EMSA (Figure 2). A SCO7658-orthologous sequence (98% nucleotide identity according to BLAST) was detected in *S. lividans*, downstream from *hyaS*, but it was not annotated as a *S. lividans* coding DNA sequence (CDS). However our microarray data suggest that this sequence is indeed a CDS or alternatively that the *S. lividans hyaS* CDS is longer than annotated.

^d^SCO genes and their *S. griseus* orthologs studied and described under another name found on StrepDB database [7] or see “References”.

^e^Fold change (Fc) in gene expression in the *S. lividans adpA* mutant with respect to the parental strain with *P*-value < 0.05, as calculated by Student’s *t*-test applying the Benjamini and Hochberg multiple testing correction. ± indicates average Fc of some gene operons (see Additional file 2: Table S2 for details).

^f^From a protein classification scheme for the *S. coelicolor* genome available from the Welcome Trust Sanger Institute database [37]: macromolecule metabolism (m. m.), small molecule metabolism (s. m.).

### Identification of new AdpA-controlled genes

To confirm that *S. lividans* AdpA controls the expression of genes identified as differentially expressed in microarray experiments, six genes were studied in more detail by qRT-PCR. The six genes were selected as having biological functions related to *Streptomyces* development or the cell envelope (*ramR*[1], *hyaS*[44] and SLI6586 [37]) or primary or secondary metabolism (SLI0755, *cchA*, and *cchB*[43]), and for having very large fold-change values (Table 1). The genes in *S. coelicolor* and *griseus* orthologous to SLI6586 and SLI6587 encode secreted proteins [12,42]. The expression levels of these genes in *S. lividans* wild-type and *adpA* strains were measured after various times of growth in liquid YEME media (Figure 1b), as shown in Figure 1a.

The *S. lividans hyaS* gene was strongly down-regulated in the *adpA* mutant compared to the wild-type (Fc < 0.03) (Figure 1b) as previously observed for the SCO0762 homolog also known as *sti1*[25]. This suggests that *hyaS* expression is strongly dependent on *S. lividans* AdpA or an AdpA-dependent regulator. SLI0755, SLI6586 and *ramR*, were also expressed at a lower level in the *adpA* mutant than wild-type, particularly after mid-exponential phase (Figure 1b, times C, D and T); *cchB* seemed to be mostly affected by AdpA during stationary phase (Figure 1b, time T). The expression of *cchA* was strongly down-regulated by the absence of AdpA at times D and T (Figure 1b): note that despite repeated efforts, *cchA* expression could not be detected in samples corresponding to times A to C for unknown reasons. The findings for gene expression as determined by microarrays and by qRT-PCR were consistent, with the exception of those for *ramR*. The expression of *ramR* observed by qRT-PCR at time T differed from that determined in microarray experiments (Table 1), suggesting that some of our microarray data are flattened. Nevertheless, these qRT-PCR experiments confirmed that the expression of the six selected genes is indeed AdpA-dependent in *S. lividans* at every growth time studied*.*

### Direct binding of AdpA to the promoter regions of *S. lividans* AdpA regulon members

To determine whether *S. lividans* AdpA directly controls these genes, we searched for potential AdpA*-*binding sites in their promoter regions *in silico*. A consensus AdpA*-*binding sequence (^5′^TGGCSNGWWY^3′^) has been established in *S. griseus*, and AdpA can bind up to five sites between positions -260 bp and +60 bp with respect to the transcriptional start point of the target gene [10]. BLAST analysis revealed that the *S. griseus* AdpA DNA-binding domain is conserved in *S. coelicolor* and *S. lividans* AdpAs (data not shown) suggesting that all three species share the same AdpA-binding consensus sequence.

The DNA sequences upstream from the *S. coelicolor ramR* and *hyaS* genes and the intergenic region between the divergently transcribed genes *cchA/cchB,* SCO0774/SCO0775 and SCO6197/SCO6198 were analyzed using PREDetector software [39] and a matrix was generated with identified *S. griseus* AdpA*-*binding sequences [10,23,25]. Between three and nine putative AdpA-binding sites were detected within the promoter region of the *S. coelicolor* genes and by analogy in orthologous *S. lividans* AdpA-dependent genes (Table 2, location with respect to translation start point). During the course of this study, the *S. lividans* 1326 genome sequence became available [24] (but not in a form suitable for analysis with PREDetector (version 1.2.3.0) [39]) and its analysis suggested that the position and composition of AdpA-binding sites were different from those predicted. The putative AdpA-binding sites of *S. lividans cchA/cchB* at -101 nt and -86 nt are GGGCCG*G*TTC and TGGCTGGAA*C*, respectively. The AdpA-binding sites located upstream of SLI0755, SLI6586, and *hyaS* differ from their *S. coelicolor* orthologs (see Table 2, changes in the location from translation start site are indicated in bracket).

**Table 2:** **AdpA-binding sites identified *in silico *** **in the promoter regions of *S. lividans *** **AdpA-dependent genes**^**a**^

***S. coelicolor *** **gene (*S. lividans *** **gene)**^**b**^	**Putative AdpA-binding site**^**c**^	**Position (bp) with respect to translation start site**^**c**^		**Strand location**^**d**^	**Scores**^**e**^	**Sites in EMSA probes**^**f**^
cchA/cchB*	TGGCCGGATT^#^	-425^#^		CS	9.30	+
	TGGCGACATT^#^	-254^#^		CS	5.19	+
	GGGCCGATTC (G^7th^)	-101		CS	4.99	+
	TGGCTCGAAT (C^10th^)	-86		NCS	6.91	+
*ramR*	GTGCCGGTTC	-464		NCS	3.37	-
	TGGCGCGAAA	-384		NCS	6.42	+
	CGGCCGAAAA	-358		NCS	5.85	+
	GGGCGGGTTC	-280		NCS	5.08	+
	TGGCCAGGAC	-279		CS	3.86	+
	GGGCGGATAA	-184		NCS	3.87	+
	TGTCGTGTTC	-95		CS	4.83	-
	CGGCGGAACA	-81		NCS	3.15	-
	TGGCCCGAAC	-30		CS	7.23	-
SCO0774/SCO0775*	CGGCGCGTTC	-268	(-226)	CS	4.25	-
(i.e. SLI0755/SLI0756)	GGACGGGAAC	-253	(-211)	NCS	3.37	+
	GGGCGCGATC	-207	(-165)	CS	4.53	+
	TGGCGCGATC	-170	(-128)	NCS	6.90	+
	CGGCCAGTCT	-110	(-68)	CS	3.06	+
	TGGCCGAACT	-84	(-42)	CS	6.20	-
	CGGCCAGATC	-79	(-37)	NCS	5.84	-
SCO6197/SCO6198*	GGTCCGGACA	-499	(-547^~^)	CS	4.98	-
(i.e. SLI6586/SLI6587)	TGACCAGAAG	-414	(-462^~^)	CS	3.82	+
	TGGCCGAGTT	-362	(-410^~^)	CS	5.06	+
	GTTCCTGCAA	-297	(-345^~^)	NCS	3.50	+
	GGGCTGAAAC	-271	(-319^~^)	NCS	4.77	+
	TGGCTGAATT	-116	(-164)	CS	7.85	+
*hyaS*	TGGCCGGATC	-130	(-129)	NCS	8.90	+
	CGGCCATTTC	-124	(-123)	CS	3.05	+
	TGTCCAGAAG	-101	(-100)	NCS	4.48	+

^a^*In silico* analysis of the *S. coelicolor* genome using PREDetector software (version 1.2.3.0, the *S. lividans* database was not available at the time this analysis was performed) [39] to analyse orthologs of *S. lividans* AdpA-dependent genes. The *S. coelicolor* AdpA-binding sites identified were checked for their conservation and location using the *S. lividans* genome StrepDB database [7] (see legend c).

^b^Genes are named according to the StrepDB database [7]. *binding sites located between *S. coelicolor* genes transcribed in the opposite orientation.

^c^Putative *S. coelicolor* AdpA-binding sites were found *in silico* with PREDetector [39]; ^#^putative site located in the upstream from the CDS of *cchB*. The site location given corresponds to the position of first nucleotide most distant from the translation start point of the first gene named. The positions of some sites are not the same for the *S. lividans* orthologs as indicated in brackets (*S. lividans* StrepDB database [7]). ^~^ putative sites are in the CDS of SLI6587. Predicted CDS diverge between SLI6586 and SLI6587 locus and their orthologs SCO6197 and SCO6198, resulting in a smaller intergenic region in *S. lividans*.

^d^CS, coding strand; NCS, non coding strand with reference to the first gene named in the *S. coelicolor* gene column.

^e^Scores given by PREDetector software for *S. coelicolor* genes [39].

^f^Sites present (+) or absent (-) in the *S. lividans* DNA probes used in EMSA experiments.

**Figure 2:**
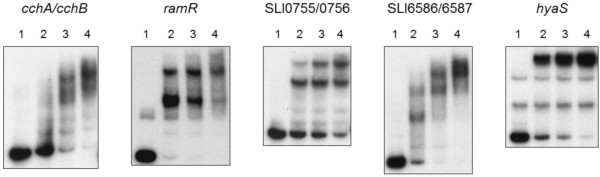
**AdpA binds *in vitro *to promoter DNA regions of *S. lividans *AdpA-dependent genes.** Electrophoretic mobility shift assays performed with 0 (lane 1), 5.7 (lane 2), 11.4 (lane 3) or 17.1 (lane 4) pmoles of purified AdpA-His_6_ and 32P-labelled probes (10,000 cpm) corresponding to the regions upstream of the *S. lividans* genes indicated, in the presence of competitor DNA (1 μg poly dI-dC).

We used EMSA to test whether *S. lividans* AdpA binds to predicted *S. lividans* AdpA-binding sequence. Recombinant purified AdpA-His_6_ bound to the promoter region of *S. lividans sti1* (SCO0762 homolog), an AdpA-dependent gene, whereas an excess of AdpA-His_6_ (up to 34 pmoles) did not bind to the promoter of SLI4380 (SCO4141 homolog), a gene that is not controlled by *S. lividans* AdpA. This suggests that the binding of AdpA with the promoter of genes tested in our previous study was specific [25]. AdpA-His_6_ was able to bind to the promoter regions of all *S. lividans* AdpA-dependent genes tested (Table 2, Figure 2), although with different affinities. For SLI6586/SLI6587, *ramR* and *hyaS*, displacement of the DNA fragment to the slower migrating protein-DNA complex was nearly complete with amounts of AdpA of less than 11 pmoles (Figure 2, lane 2). For *cchA/cchB* and SLI0755/SLI0756, larger amounts of AdpA were necessary for near complete displacement of the DNA probe to a protein-DNA complex. In a competition EMSA performed on SLI6586/6587 with an excess of the corresponding unlabelled probe, AdpA-binding to the labelled probe decreased (data not shown). We also tested a *hyaS* promoter in which one (highest score) of the three putative AdpA-binding sites was mutated (at position -134 to -129, see Additional file 3: Figure S1a): the affinity of AdpA for this promoter region was reduced and one protein-DNA complex disappeared (Additional file 3: Figure S1b). These results suggest that one dimer of AdpA binds the adjacent sites -129 and -123 of *S. lividans hyaS* promoter and another dimer binds the -100 site resulting in the formation of the two DNA-AdpA complexes depicted in Figure 2.

These EMSA experiments demonstrated that *S. lividans* AdpA directly binds to five intergenic regions and confirmed the *in silico* prediction presented in Table 2. *S. lividans* AdpA directly regulates at least the six AdpA*-*dependent genes listed above and identified by microarrays and qRT-PCR analysis. These newly identified targets highlight the pleiotropic role of *S. lividans* AdpA: it is involved in primary (SLI0755) and secondary (*cchA*, *cchB* and *hyaS*) metabolisms, in regulation (*ramR*), and in cell development (*hyaS*, *ramR* and SLI6586).

## Discussion

AdpA, a transcriptional regulator of the AraC/XylS family, is involved in the development and differentiation of various *Streptomyces*[3-5,25]. We report here the first identification of several pathways directly regulated by AdpA in *S. lividans* cultivated in liquid rich medium.

Inactivation of *adpA* in *S. lividans* affected the expression of approximately 300 genes. This large number was expected in the light of the size of the *S. griseus* AdpA regulon [14]. Although *adpA* mutant growth was comparable to that of the parental strain in YEME liquid medium, the expression of around 200 genes involved in primary metabolism was influenced by *adpA* deletion. These genes encode proteins involved in the major biosynthesis pathways for amino acids (class 3.1. in Additional file 2: Table S2) [37], and in energy metabolism (class 3.5.) including glycolysis, pentose phosphate, pyruvate dehydrogenase pathways, as well as in electron transport (e.g. CydAB cytochrome oxidase, CYP105D5 and Fdx4 involved in fatty acid hydroxylation and encoded by SLI0755-0754 [45]). Other *S. lividans* AdpA-regulated genes influence *Streptomyces* development on solid media (e.g. those for RamR, chaplins Chp, BldN, WblA, WblE, HyaS and ClpP1ClpP2 peptidases) (Table 1) [1,6,16,25,44]. *S. lividans* AdpA also influences the expression of 18 genes involved in secondary metabolism such as coelichelin biosynthesis (*cch* genes in Table 1) [43] and also genes described to affect metabolic differentiation (HyaS, CutRS, WblA, DesE, and CdtCBA) (Table 1) [15,17,42,44]. Consistently with transcriptomic studies in *S. griseus*, these observations suggest that AdpA is a pleiotropic transcriptional regulator in *S. lividans*.

We demonstrate that *S. lividans* AdpA directly activates *cchB*, SLI0755 and *hyaS.* As a result of their co-transcription with these genes, the expression of *cchCD*, SLI0754 and SCO7658-ortholog genes is AdpA-dependent in *S. lividans* (Table 1). SLI0756 is probably a directly AdpA-regulated gene because its promoter DNA region is shared with SLI0755-SLI0754 operon, which is transcribed in the opposite direction and directly regulated by AdpA (Table 1, Figure 2).

AdpA directly regulates the genes *ramR* and *sti1* in *S. lividans* (this study) [25] and in the closely related species *S. coelicolor*[16]. In an *S. coelicolor adpA* mutant, levels of *sti1* and *ramR* expression were lower than in the wild-type strain following growth for 48 h in a minimal agar medium [16]. *In vitro* experiments showed a high affinity of AdpA with a *S. coelicolor sti1* probe [16], consistent with our results with *S. lividans sti1*[25]. However, AdpA had a lower affinity to *S. coelicolor ramR* (with promoter region -302 nt to +73 nt with respect to the translation start site) than *S. lividans ramR* (Figure 2, with the promoter region -440 nt to -181 nt). When we used a *S. lividans ramR* probe carrying the promoter region from -201 nt to +66 nt, we observed that less than half the probe was shifted (data not shown). Therefore, the predicted sites for *ramR* promoter at positions -384 and -358 (Table 2) may have the greatest affinity for AdpA (Figure 2). Of the genes analysed by qRT-PCR, the *ramR* gene was that for which the observed expression was the least consistent with the microarray findings, even through the same sample was used for these analyses. This suggests that the expression of genes close to the cut-off we applied to the microarray data will need further investigation by qRT-PCR.

Among the 28 genes identified as direct targets of AdpA in *S. griseus*, 13 have no orthologous gene in *S. lividans* and the orthologous genes of six are not under the control of *S. lividans* AdpA in our conditions. In addition to *ramR* (*amfR*) and *sti1* (*sgiA*), *hyaS* (SGR3840) is also a directly AdpA-regulated gene that is conserved in the *S. lividans* and *S. griseus* AdpA regulons [12,25]. In *S. lividans*, *hyaS* affects hypha aggregation and the amount of mycelium-associated undecylprodigiosin [44]; its function in *S. griseus* is unknown. The expression of all of *bldN*, SLI6392, SLI1868 and the SCO2921 ortholog (gene detected in *S. lividans* genome but not named in StrepDB [7]) is influenced by *adpA* deletion in *S. lividans*. It remains to be determined whether AdpA directly controls *S. lividans adpA* and *bldA* as described in *S. coelicolor* and *griseus*[16,23].

*S. coelicolor adpA* is one of 145 identified TTA-containing genes; the production of the proteins encoded by these genes is dependent on *bldA*, encoding the only tRNA for the rare leucine codon TTA [46]. Our study has revealed that expression of 11 TTA-containing genes and of 24 genes regulated by *S. coelicolor bldA*[42,47,48] was affected by *adpA* deletion in *S. lividans* (Additional files 4: Table S3). We show that *cchA, cchB*, *sti1*, *hyaS*, SLI6586 and SLI6587, previously identified in *S. coelicolor* as *bldA-*dependent genes, are direct targets of *S. lividans* AdpA [25]. Of the 29 other *bldA*-dependent genes, 19 are probable direct *S. lividans* AdpA targets: *in silico* analysis indicated the presence of putative AdpA-binding sites upstream from these genes (most of them with score above 4, see Additional file 5: Table S4). By analogy, this suggests that the deregulation of certain genes observed in the *S. coelicolor bldA* mutant may have been the consequence of *S. coelicolor* AdpA down-regulation, as previously suggested [49].

To predict probable direct targets of AdpA in *S. lividans* and contribute to knowledge of the AdpA regulon, we carried out *in silico* analysis of the entire *S. coelicolor* genome using PREDetector [39], and also restricted to the *S. lividans* genes identified as being AdpA-dependent (see Additional file 5: Table S4 and Table 3). We identified 95 genes probably directly activated by *S. lividans* AdpA and 67 genes that could be directly repressed (Additional file 5: Table S4). Most of the putative AdpA-binding sites identified by this analysis are coherent with the findings of Yao et al., demonstrating the importance of G and C nucleotides at positions 2 and 4, respectively [50]. Six genes have been identified as directly regulated by AdpA in other species (*adpA*, *bldN*, *wblA,* SLI6392, SCO2921 orthologs, and *glpQ1*, as indicated in Table 3 in bold) [10,12,15,16,18], and 27 more in *S. griseus* are also probable AdpA-direct targets (e.g. *cchB*, SLI0755-0754 operon, *rarA* operon, *scoF4, groEL1*, SLI6587, SLI4345, *cydAB*, and *ectABD*, as indicated in Table 3 and Additional file 2: Table S2, underlined) [7,12-14]. Sixty-three of the 162 probable direct targets of AdpA in *S. lividans* have no ortholog in the *S. griseus* genome (Additional file 5: Table S4).

**Table 3:** **Genes putatively directly regulated by *S. lividans *** **AdpA in liquid rich medium**^**a**^

**Gene**^**b**^	**Gene**^**b**^	**Gene**^**b**^	**Gene name**^**b**^	**cis-element**^**c**^	**Score**^**c**^	**Position**^**c**^	**Fc**^**d**^	**Class**^**e**^
Probably directly activated by *S. lividans* AdpA:								
SCO2921*	Detected	**SGR4618**	***adbS3-orfa***	tttgcggaca	4.62	-260	0.196	c. e.
SCO0494	SLI0454	SGR6714	*cchF*	tgtcgcgcca	4.36	-28	0.615	s. m.
SCO0929	SLI1160	*SGR710*		tggccggacg	5.19	-201	0.419	u. f.
SCO1565	SLI1668	**SGR5973**	***glpQ1***	cggccggaac	6.75	-82	0.531	c. e.
SCO1630	SLI1934	*SGR1063*	*cvn9, rarA*	tgtcgggatc	6.71	-74	0.505	c. e.
SCO1674	SLI1979	SGR5829	*chpC*	cggcggaatc	5.69	-154	0.564	c. e.
SCO1800	SLI2108	SGR5696	*chpE*	cggccggacc	4.69	-65	0.256	c. e.
SCO1968	SLI2284	SGR5556	*glpQ2*	cattcagcct	3.75	-92	0.537	m. m.
**SCO2792**	SLI3139	**SGR4742**	***adpA bldH***	gaaccggcca	8.09	-148	0.383	r.
**SCO3323**	SLI3667	**SGR4151**	***bldN, adsA***	gttccggtca	6.38	-469	0.389	r.
SCO3579*	SLI3822	*SGR3340*	*wblA*	tggcccgaac	7.23	-135	0.31	r.
SCO3917*	SLI4175	*SGR3663*		ctttcggcca	6.52	-72	0.504	u. f.
SCO4113	SLI4344	*SGR3901*		aaacccgtca	5.64	-52	0.568	u. f.
SCO4114*	SLI4345	*SGR3902*		tggcgggatt	8.66	-117	0.487	c. p.
SCO4164	SLI4405	*SGR3965*	*cysA*	gttgccgcca	5.70	-170	0.483	s. m.
SCO4295*	SLI4532	*SGR3226*	*scoF4*	attctcgcca	7.13	-193	0.217	c. p.
SCO4761	SLI5031	*SGR2770*	*groES*	aaccccgccg	3.31	-197	0.401	c. p.
SCO4762	SLI5032	*SGR2769*	*groEL1*	ttgccgtata	4.40	-44	0.44	c. p.
SCO4768	SLI5039	SGR2759	*bldM*	aatctagccg	5.52	-292	0.586	r.
SCO5101	SLI5379	*SGR2456*		cggcgggaac	6.11	-28	0.584	u. f.
SCO6004	SLI6392	**SGR1503**		cggccgcatt	5.21	-292	0.603	c. e.
SCO6096*	SLI6490	*SGR1397*		catcgcgcca	5.56	-147	0.557	c. e.
SCO7550	SLI7772	-	*glpQ3*	gaaccggtca	5.88	-117	0.334	c. e.
Probably directly repressed by *S. lividans* AdpA:								
SCO1684	SLI1989	*SGR5819*		gaatgcgcca	5.36	-161	1.626	u. f.
SCO1776*	SLI2080	*SGR5721*	*pyrG*	cttccggcca	7.25	-170	1.744	s. m.
SCO1821	SLI2130	*SGR5674*	*moaA*	cggcccgaac	5.39	-61	1.679	s. m.
SCO1864	SLI2175	*SGR5635*	*ectA*	atttcggaca	6.71	-203	2.903	c. p.
SCO1865	SLI2176	*SGR5634*	*ectB*	cggccgggac	3.24	-78	3.154	c. p.
SCO1867	SLI2178	*SGR5632*	*ectD*	gaagtggcca	4.62	-3	3.029	n. c.
SCO3123	SLI3480	*SGR4383*	*prsA2*	tgaccggaaa	6.21	#	1.891	s. m.
SCO3202	SLI3556	*SGR4276*	*hrdD*	aatccggaca	7.75	-145	2.499	r.
SCO3811	SLI4062	*SGR3768*	*dacA*	tatccggacg	5.34	-175	1.628	c. e.
SCO3945	SLI4193	*SGR3646*	*cydA*	tgtcccgatt	6.39	-88	3.386	s. m.
SCO3947	SLI4195	SGR3644	*cydCD*	catcccgccg	5.08	-30	2.653	s. m.
SCO3971	SLI4220	*SGR3620*		tggccggtac	7.78	-465	1.631	u. f.
SCO4215	SLI4452	-	*xlnR*	gatgaggccg	3.74	-294	1.964	r.
SCO5240	SLI5531	SGR2274	*wblE*	tgtcccgatc	5.99	-170	2.246	u. f.
SCO5862	SLI6134	SGR1670	*cutR*	tggccgaaaa	7.69	-99	1.927	r.
SCO6009	SLI6398	*SGR1498*		cttccagcca	6.53	-52	1.736	c. p.

^a^Orthologs of *S. lividans* AdpA-dependent genes (listed in Additional file 2: Table S2) were analysed *in silico* using the *S. coelicolor* genome database (version 1.2.3.0 of PREDetector software [39]). AdpA-binding sites upstream from *S. coelicolor* genes were identified and are presented in Additional file 5: Table S4. Table 3 presents a selected subset of this complete compilation.

^b^Gene names for *S. griseus* (SGR) and annotated function are from the StrepDB database [7]. Ortholog gene names were identified using StrepDB. Genes identified in other *Streptomyces* as being directly AdpA-regulated are in bold, and those described as being AdpA-dependent are italicized [12-15,22]. * Binding sites in the promoters of these genes were identified *in silico*[22]. The SCO2921-ortholog was not annotated as a *S. lividans* CDS; however, our microarray data suggest that this CDS exists.

^c^cis-element, score, and binding site position as determined by analysing *S. coelicolor* genes with PREDetector [39]. When more than one putative AdpA-binding site was detected, only the one with the highest score was shown here. Other genes putatively directly regulated by *S. lividans* AdpA are listed in Additional file 5: Table S4. # site found in the SCO3122 CDS at position 1447 (total gene length 1449 nt).

^d^Fold change (Fc) in gene expression in *S. lividans adpA* mutant relative to the parental strain with *P*-value < 0.05, as determined by Student’s *t*-test applying the Benjamini and Hochberg multiple testing correction (details in Additional file 2: Table S2).

^e^From a protein classification scheme for the *S. coelicolor* genome available on the Welcome Trust Sanger Institute database [37]: unknown function (u. f.), cell process (c. p.), macromolecule metabolism (m. m.), small molecule metabolism (s. m.), cell envelope (c. e.), extrachromosomal (e.), regulation (r.) and not classified (n. c.).

## Conclusions

In conclusion, this study has extended our knowledge of the *S. lividans* AdpA regulon. We identified *S. lividans* AdpA-regulated genes by transcriptomic analysis, and used *in silico* analysis to identify over a hundred probable direct targets of AdpA in *S. lividans.* Most of them are absent from the current predicted *S. griseus* AdpA regulon. Discovering new *S. lividans* genes directly regulated by AdpA and that are involved in primary and secondary metabolism will provide valuable information about *Streptomyces* development and differentiation in liquid culture.

## Availability of supporting data

Microarray data are available in the ArrayExpress database [51,52] under accession number A-MEXP-2383.

## Abbreviations

qRT-PCR: Quantitative real-time PCR; ORF: Open reading frame; Fc: Fold change; CT: Critical threshold cycle; BLAST: Basic local alignment search tool; EMSA: Electrophoretic mobility shift assay; AdpA-His6: Recombinant AdpA protein with a six-histine tag at the C-terminus; CDS: Coding DNA sequence; CS: Coding strand; NCS: Non coding strand; u. f.: Unknown function; c. p.: Cell process; m. m.: Macromolecule metabolism; s. m.: Small molecule metabolism; c. e.: Cell envelope; e.: Extrachromosomal; r.: Regulation; n. c.: Not classified.

## Competing interests

The authors declare that they have no competing interests.

## Authors’ contributions

AG, NB and PM wrote and revised the manuscript. CP and JYC have given final approval for this version to be published. PM helped AG to design the project. AG performed qRT-PCR, EMSA and *in silico* analysis; and prepared Figures, Tables and Additional files. NB purified AdpA-His_6_ protein. CP carried out the microarray experiments. JYC helped CP with the statistical analysis of microarray results and wrote the associated Methods sections. AG interpreted the microarrays data. MG help with qRT-PCR experiments and provided technical support. All authors read and approved the final manuscript.

## Authors’ information

AG performed qRT-PCR and EMSA experiments while working at Pasteur Institute. Her current address is Centre for Bacterial Cell Biology, Institute for Cell and Molecular Biosciences, Newcastle University, Newcastle-upon-Tyne NE2 4HH, UK.

## Supplementary Material

Additional file 1: Table S1 Oligonucleotides used in this study. Click here for file

Additional file 2: Table S2 Complete set of genes differentially expressed in the *S. lividans adpA* mutant. *S. coelicolor* microarrays were used to test for genes differentially expressed in the *S. lividans adpA* mutant and wild-type 1326, at growth time point T, in liquid YEME medium. Annotated function, Fc, *P*-values, and classification of the proteins are presented according to the microarray SCO genes, by increasing SCO gene number. Click here for file

Additional file 3: Figure S1 Effect of the mutation of one AdpA-binding site in the *S. lividans hyaS* promoter on AdpA-binding specificity. Mutation of an AdpA-binding site in the *S. lividans hyaS* promoter region prevents formation of an AdpA-DNA complex *in vitro*. Sequence of the mutated AdpA-binding site (at -129 nt) and EMSA performed with the mutated *hyaS* promoter region are shown. Click here for file

Additional file 4: Table S3 Comparison of gene expression profiles between *S. coelicolor bldA*-dependent and *S. lividans* AdpA-dependent genes. Comparison of the gene expression profiles of some *S. coelicolor bldA*-dependent genes whose *S. lividans* orthologs are AdpA-dependent (see Additional file 2: Table S2). Putative AdpA-binding sites were identified *in silico* (see Additional file 5: Table S4), suggesting that in the *S. coelicolor bldA* mutant*,* the *adpA* translation defect leads to *bldA*-dependence of the genes identified previously [42,47,48]. Click here for file

Additional file 5: Table S4 Putative *S. coelicolor* AdpA-binding sites upstream from the *S. lividans* AdpA-dependent genes. We identified putative AdpA-binding sites *in silico* using the *S. coelicolor* genome and we analysed orthologs of *S. lividans* AdpA-dependent genes (based on our microarray data); the sequences and positions of the sites with the highest scores according to PREDetector are shown. *S. coelicolor*, *S. lividans* and *S. griseus* ortholog genes are indicated and previously identified direct or probably direct *S. griseus* AdpA-dependent genes are highlighted. Click here for file
