# Supplementary material for: Identified members of the Streptomyces lividans AdpA regulon involved in differentiation and secondary metabolism
Source: BMC Microbiol. 2014 Apr 3;14:81. doi: 10.1186/1471-2180-14-81 (PMC4021200; doi:10.1186/1471-2180-14-81)
Supplement: Additional file 1: Table S1 — Oligonucleotides used in this study. [file 1471-2180-14-81-S1.pdf]

Additional file 1 – Oligonucleotides used in this study

Oligonucleotide	Sequence (5' to 3')
RTcchA-1	ACATCATCGTCGCCAACAACTG
RTcchA-2	ACGCCCACCTCGGTCTCG
RTcchB-1	CAGGTTCTTCCTGCTGAC
RTcchB-2	GCCTGTGCGTTGTGTTCG
RTsli0755-1	GACCGACTGGAGGCGTACCTG
RTsli0755-2	GTTGGTCAGGAGGGTGTAGG
RTsli6586-1	CAAGAGCGGAACCAATCAG
RTsli6586-2	TGAGTGTTGAAGTCGTTGC
RTramR-1	TTGCGGGCACCCACCTTC
RTramR-2	GCTGACCACGAGGGAACTG
RThyaS-1	GCGACCACCTCACTCACGCTC
RThyaS-2	CTTGGCCTTGCCGCCCTC
MG16-hrdB	CCTCCGCCTGGTGGTCTC
MG17-hrdB	AGAACTTGTAGCCCTTGGTGTAG
GScchA-1	GTTCCTCCTCAGGGCAGGGGCT
GScchA-2	GAAGAGGTTCTTGTGGTTGATGAAGT
GSsli0755-1	GTTCCCGTCCTTACCGCAT
GSsli0755-2	CAGATCGGTGGCGGAGGC
GSsli6586-1	GCTGGCTATCCATACGAAGATCCATTG
GSsli6586-2	CTCAGAAAGGAAGCGACGCGCACGTATGA
GSramR-3	GACTAGGGGCACCCACCGACAGCCA
GSramR-4	GAAGGGCGGATAACACACCAATC
GShyaS-1	GAACTGCCGCTCAGCCGTCA
GShyaS-2	GAAGGAAGCAGGCGGGCACT
