# Supplementary material for: Identified members of the Streptomyces lividans AdpA regulon involved in differentiation and secondary metabolism
Source: BMC Microbiol. 2014 Apr 3;14:81. doi: 10.1186/1471-2180-14-81 (PMC4021200; doi:10.1186/1471-2180-14-81)
Supplement: Additional file 2: Table S2 — Complete set of genes differentially expressed in the S. lividans adpA mutant. S. coelicolor microarrays were used to test for genes differentially expressed in the S. lividans adpA mutant and wild-type 1326, at growth time point T, in liquid YEME medium. Annotated function, Fc, P-values, and classification of the proteins are presented according to the microarray SCO genes, by increasing SCO gene number. [file 1471-2180-14-81-S2.pdf]

Additional file 2 – Complete set of genes differentially expressed in the *S. lividans adpA* mutant[a].

gene[b]	annotated function[b]	Fc[c]	*P*-value[d]	class[e]	classification details[e]
SCO0148	putative transcriptional regulatory protein	0.421	0.00E+00	r.	6.5.0 Others (237)
SCO0169	conserved hypothetical protein SCJ1.18	0.621	2.70E-08	u. f.	0.0.2 Conserved in organism other than Escherichia coli (1322)
SCO0171	conserved hypothetical protein SCJ1.20	0.615	1.30E-08	u. f.	0.0.2 Conserved in organism other than Escherichia coli (1322)
SCO0188	putative methylesterase	0.531	4.90E-13	s. m	3.8.0 Secondary metabolism (165)
SCO0197	conserved hypothetical protein	1.820	1.29E-11	u. f.	0.0.2 Conserved in organism other than Escherichia coli (1322)
SCO0199	putative alcohol dehydrogenase	1.927	0.00E+00	s. m	3.5.2 Anaerobic respiration (22)
SCO0200	conserved hypothetical protein SCJ12.12c	1.656	2.53E-09	u. f.	0.0.2 Conserved in organism other than Escherichia coli (1322)
SCO0205	putative pyruvate formate-lyase activating enzyme (pseudogene)	1.684	5.42E-10	s. m	3.5.2 Anaerobic respiration (22)
SCO0216	nitrate reductase alpha chain NarG2	1.745	1.86E-11	s. m	3.5.2 Anaerobic respiration (22)
SCO0218	putative nitrate reductase delta chain NarJ2	1.650	8.44E-07	s. m	3.5.2 Anaerobic respiration (22)
SCO0224	conserved hypothetical protein	1.782	2.14E-12	u. f.	0.0.2 Conserved in organism other than Escherichia coli (1322)
SCO0229	putative oxidoreductase (putative secreted protein)	1.988	0.00E+00	c. e.	4.1.6 Gram positive membrane (836)
SCO0231	small hydrophobic hypothetical protein	0.122	0.00E+00	u. f.	0.0.0 Unknown function. no known homologs (1049)
SCO0268	hypothetical protein	0.100	0.00E+00	u. f.	0.0.0 Unknown function. no known homologs (1049)
SCO0280	conserved hypothetical protein SCF85.08c	0.332	0.00E+00	u. f.	0.0.2 Conserved in organism other than Escherichia coli (1322)
SCO0338	putative dehydrogenase	1.865	0.00E+00	s. m	3.5.6 Oxidative branch. pentose pwy (9)
SCO0370	possible DNA-binding protein (putative secreted protein)	0.575	2.38E-08	r.	6.5.0 Others (237)
SCO0379	catalase (EC 1.11.1.6)	0.483	0.00E+00	c. p.	1.4.2 Detoxification (90)
SCO0382	UDP-glucose/GDP-mannose family dehydrogenase	0.491	0.00E+00	s. m	3.8.0 Secondary metabolism (165)
SCO0383	hypothetical protein SCF62.09	0.527	0.00E+00	s. m	3.8.0 Secondary metabolism (165)
SCO0384	putative membrane protein	0.611	7.01E-09	s. m	3.8.0 Secondary metabolism (165)
SCO0391	putative transferase	0.613	3.94E-03	s. m	3.8.0 Secondary metabolism (165)
SCO0392	putative methyltransferase	0.606	1.24E-05	s. m	3.8.0 Secondary metabolism (165)
SCO0394	hypothetical protein SCF62.20	0.518	0.00E+00	s. m	3.8.0 Secondary metabolism (165)
SCO0396	hypothetical protein SCF62.22	0.454	0.00E+00	s. m	3.8.0 Secondary metabolism (165)
SCO0397	putative integral membrane protein	0.312	0.00E+00	s. m	3.8.0 Secondary metabolism (165)
SCO0399	putative membrane protein	0.532	0.00E+00	s. m	3.8.0 Secondary metabolism (165)
SCO0402	putative integral membrane protein	0.578	4.02E-11	c. e.	4.1.6 Gram positive membrane (836)
SCO0423	putative membrane transport protein	0.621	3.44E-07	c. p.	1.5.0 Transport/binding proteins (520)
SCO0438	pyrrolidone-carboxylate peptidase (EC 3.4.19.3)	1.605	6.06E-04	m. m.	2.1.4 Degradation of proteins. peptides. glycoproteins (126)
SCO0439	putative membrane protein	1.840	1.10E-13	c. e.	4.1.6 Gram positive membrane (836)
SCO0453	probable secreted solute-binding lipoprotein.	1.876	5.78E-11	c. p.	1.5.0 Transport/binding proteins (520)
SCO0464	putative integral membrane protein	1.723	6.14E-11	c. e.	4.1.6 Gram positive membrane (836)
SCO0494	putative iron-siderophore binding lipoprotein	0.615	1.26E-08	s. m	3.8.0 Secondary metabolism (165)
SCO0496	putative iron-siderophore permease transmembrane protein	0.505	0.00E+00	s. m	3.8.0 Secondary metabolism (165)
SCO0497	putative iron-siderophore permease transmembrane protein	0.492	0.00E+00	s. m	3.8.0 Secondary metabolism (165)
SCO0498	putative peptide monooxygenase	0.336	0.00E+00	s. m	3.8.0 Secondary metabolism (165)
SCO0499	putative formyltransferase	0.374	0.00E+00	s. m	3.8.0 Secondary metabolism (165)
SCO0560	catalase/peroxidase	0.565	4.51E-12	c. p.	1.4.2 Detoxification (90)

Additional file 2 – Complete set of genes differentially expressed in the *S. lividans adpA* mutant[a].

gene[b]	annotated function[b]	Fc[c]	*P*-value[d]	class[e]	classification details[e]
SCO0561	Fe regulatory protein	0.613	9.49E-09	r.	6.5.0 Others (237)
SCO0592	hypothetical protein SCF55.16c	0.624	4.10E-08	u. f.	0.0.0 Unknown function. no known homologs (1049)
SCO0611	putative membrane protein	1.682	6.22E-10	c. e.	4.1.6 Gram positive membrane (836)
SCO0643	putative secreted cellulose-binding protein.	2.002	0.00E+00	c. p.	1.5.3 Carbohydrates. organic acids and alcohols (9)
SCO0682	hypothetical protein SCF15.03c	0.375	0.00E+00	u. f.	0.0.0 Unknown function. no known homologs (1049)
SCO0705	conserved hypothetical protein	0.579	4.55E-11	u. f.	0.0.2 Conserved in organism other than Escherichia coli (1322)
SCO0762	protease inhibitor precursor	0.124	0.00E+00	m. m.	2.1.4 Degradation of proteins. peptides. glycoproteins (126)
SCO0771	conserved hypothetical protein	0.612	1.66E-06	u. f.	0.0.2 Conserved in organism other than Escherichia coli (1322)
SCO0772	putative regulatory protein	0.618	1.81E-08	r.	6.3.8 TetR (122)
SCO0773	putative ferredoxin	0.098	0.00E+00	s. m	3.5.3 Electron transport (71)
SCO0774	putative cytochrome P450	0.075	0.00E+00	s. m	3.5.3 Electron transport (71)
SCO0775	conserved hypothetical protein	0.424	0.00E+00	u. f.	0.0.2 Conserved in organism other than Escherichia coli (1322)
SCO0827	hypothetical protein SCF43A.17c	0.516	0.00E+00	u. f.	0.0.0 Unknown function. no known homologs (1049)
SCO0860	probable cation-transporting ATPase	0.526	0.00E+00	c. p.	1.5.2 Cations (40)
SCO0862	putative integral membrane protein.	0.243	0.00E+00	c. e.	4.1.6 Gram positive membrane (836)
SCO0863	putative integral membrane protein	0.225	0.00E+00	c. e.	4.1.6 Gram positive membrane (836)
SCO0864	probable ECF-family sigma factor.	0.574	1.97E-11	r.	6.2.1 sigma factor (66)
SCO0865	putative integral-membrane protein.	0.621	2.97E-08	c. e.	4.1.6 Gram positive membrane (836)
SCO0919	hypothetical protein SCM10.07c	0.621	2.94E-08	u. f.	0.0.0 Unknown function. no known homologs (1049)
SCO0920	putative acyltransferase	0.503	0.00E+00	m. m.	2.2.7 Phospholipids (14)
SCO0923	putative reductase flavoprotein subunit	1.752	1.23E-11	s. m	3.5.8 TCA cycle (26)
SCO0928	conserved hypothetical protein SCM10.16c	0.584	1.14E-10	u. f.	0.0.2 Conserved in organism other than Escherichia coli (1322)
SCO0929	conserved hypothetical protein SCM10.17c	0.419	0.00E+00	u. f.	0.0.2 Conserved in organism other than Escherichia coli (1322)
SCO1032	putative ABC transport system ATP-binding protein	2.138	0.00E+00	c. p.	1.5.0 Transport/binding proteins (520)
SCO1048	putative secreted protein	0.311	0.00E+00	c. e.	4.1.7 Gram positive exported/lipoprotein (439)
SCO1088	putative oxidoreductase	1.701	2.09E-10	n. c.	7.0.0 Not classified (included putative assignments) (565)
SCO1132	putative oxidoreductase	0.559	2.83E-09	n. c.	7.0.0 Not classified (included putative assignments) (565)
SCO1134	putative oxidoreductase. iron-sulphur binding subunit	0.584	1.06E-10	n. c.	7.0.0 Not classified (included putative assignments) (565)
SCO1155	conserved hypothetical protein	0.608	4.34E-09	u. f.	0.0.2 Conserved in organism other than Escherichia coli (1322)
SCO1179	conserved hypothetical protein SCG11A.10c	1.649	3.71E-09	u. f.	0.0.2 Conserved in organism other than Escherichia coli (1322)
SCO1183	conserved hypothetical protein SCG11A.14	1.682	6.10E-10	u. f.	0.0.2 Conserved in organism other than Escherichia coli (1322)
SCO1184	conserved hypothetical protein SCG11A.15	1.611	2.75E-08	u. f.	0.0.2 Conserved in organism other than Escherichia coli (1322)
SCO1186	putative lacI-family transcriptional regulator	1.657	2.42E-09	r.	6.3.5 LacI (34)
SCO1196	putative secreted protein	0.493	0.00E+00	c. e.	4.1.7 Gram positive exported/lipoprotein (439)
SCO1222	conserved hypothetical protein	0.509	0.00E+00	u. f.	0.0.2 Conserved in organism other than Escherichia coli (1322)
SCO1223	ornithine aminotransferase	0.617	1.77E-08	s. m	3.1.2 Arginine (10)
SCO1269	putative pyruvate dehydrogenase beta subunit	1.752	1.22E-11	s. m	3.8.1 PKS (102)
SCO1282	putative oxidoreductase	1.620	1.77E-08	s. m	3.8.0 Secondary metabolism (165)
SCO1361	conserved hypothetical protein	0.407	0.00E+00	u. f.	0.0.2 Conserved in organism other than Escherichia coli (1322)
SCO1379	hypothetical protein	1.800	7.80E-13	u. f.	0.0.2 Conserved in organism other than Escherichia coli (1322)

Additional file 2 – Complete set of genes differentially expressed in the *S. lividans adpA* mutant[a].

gene[b]	annotated function[b]	Fc[c]	*P*-value[d]	class[e]	classification details[e]
SCO1407	hypothetical protein	0.617	2.13E-07	u. f.	0.0.0 Unknown function. no known homologs (1049)
SCO1430	putative TetR-family transcriptional regulator.	1.685	5.26E-10	r.	6.3.8 TetR (122)
SCO1435	putative integral membrane protein	1.743	2.01E-11	c. e.	4.1.6 Gram positive membrane (836)
SCO1444	putative chitinase precursor	1.984	0.00E+00	m. m.	2.1.3 Degradation of polysaccharides (92)
SCO1499	putative integral membrane protein	1.659	2.21E-09	c. e.	4.1.6 Gram positive membrane (836)
SCO1565	putative glycerophosphoryl diester phosphodiesterase	0.531	0.00E+00	c. e.	4.1.7 Gram positive exported/lipoprotein (439)
SCO1593	putative transcriptional regulator	1.860	0.00E+00	r.	6.3.2 AraC (33)
SCO1626	putative cytochrome P450	0.518	0.00E+00	s. m	3.5.3 Electron transport (71)
SCO1627	putative ATP-GTP binding protein	0.423	0.00E+00	n. c.	7.0.0 Not classified (included putative assignments) (565)
SCO1628	conserved hypothetical protein	0.414	0.00E+00	u. f.	0.0.2 Conserved in organism other than Escherichia coli (1322)
SCO1629	conserved hypothetical protein SCI41.12c	0.375	0.00E+00	u. f.	0.0.2 Conserved in organism other than Escherichia coli (1322)
SCO1630	putative integral membrane protein	0.505	0.00E+00	c. e.	4.1.6 Gram positive membrane (836)
SCO1674	putative secreted protein	0.564	3.74E-12	c. e.	4.1.7 Gram positive exported/lipoprotein (439)
SCO1675	putative small membrane protein	0.237	0.00E+00	c. e.	4.1.6 Gram positive membrane (836)
SCO1684	hypothetical protein	1.626	1.68E-07	u. f.	0.0.0 Unknown function. no known homologs (1049)
SCO1688	putative membrane protein	1.609	3.10E-08	c. e.	4.1.6 Gram positive membrane (836)
SCO1700	putative membrane protein	0.524	0.00E+00	c. e.	4.1.6 Gram positive membrane (836)
SCO1715	putative homogentisate 1.2-dioxygenase	0.601	1.65E-09	s. m	3.4.2 Amino acids (28)
SCO1769	conserved hypothetical protein	1.619	1.85E-08	u. f.	0.0.2 Conserved in organism other than Escherichia coli (1322)
SCO1776	putative CTP synthetase	1.744	1.93E-11	s. m	3.3.11 Nucleotide interconversions (25)
SCO1800	putative small secreted protein	0.256	0.00E+00	c. e.	4.1.7 Gram positive exported/lipoprotein (439)
SCO1821	molybdenum cofactor biosynthesis protein A	1.679	7.20E-10	s. m	3.2.9 Molybdopterin (8)
SCO1860	putative secreted protein	0.344	0.00E+00	c. e.	4.1.7 Gram positive exported/lipoprotein (439)
SCO1864	putative acetyltransferase	2.903	0.00E+00	c. p.	1.6.2 Osmotic adaptation (7)
SCO1865	putative aminotransferase	3.154	0.00E+00	c. p.	1.6.2 Osmotic adaptation (7)
SCO1866	putative condensing enzyme	2.552	0.00E+00	c. p.	1.6.2 Osmotic adaptation (7)
SCO1867	putative hydroxylase	3.029	0.00E+00	n. c.	7.0.0 Not classified (included putative assignments) (565)
SCO1887	putative integral membrane transport protein	1.656	2.56E-09	c. p.	1.5.0 Transport/binding proteins (520)
SCO1968	putative secreted hydrolase	0.537	0.00E+00	m. m.	2.2.7 Phospholipids (14)
SCO1969	putative DNA-methyltransferase	0.564	3.48E-12	m. m.	2.2.3 DNA - replication. repair. restr./modific'n (85)
SCO2068	putative secreted alkaline phosphatase	0.621	2.77E-08	c. e.	4.1.7 Gram positive exported/lipoprotein (439)
SCO2174	putative transferase	1.819	3.00E-13	n. c.	7.0.0 Not classified (included putative assignments) (565)
SCO2180	putative dihydrolipoamide dehydrogenase	1.784	1.88E-12	s. m	3.5.7 Pyruvate dehydrogenase (10)
SCO2181	putative dihydrolipoamide succinyltransferase	1.713	1.09E-10	s. m	3.5.5 Glycolysis (25)
SCO2183	putative pyruvate dehydrogenase E1 component	1.647	4.20E-09	s. m	3.5.7 Pyruvate dehydrogenase (10)
SCO2212	hypothetical protein SC10B7.07	0.516	0.00E+00	u. f.	0.0.0 Unknown function. no known homologs (1049)
SCO2286	putative alkaline phosphatase	0.623	3.62E-08	n. c.	7.0.0 Not classified (included putative assignments) (565)
SCO2289	putative membrane protein.	0.621	2.71E-08	c. e.	4.1.6 Gram positive membrane (836)
SCO2362	hypothetical protein	1.797	9.60E-13	u. f.	0.0.0 Unknown function. no known homologs (1049)
SCO2396	conserved hypothetical protein	1.734	3.42E-11	u. f.	0.0.2 Conserved in organism other than Escherichia coli (1322)

Additional file 2 – Complete set of genes differentially expressed in the *S. lividans adpA* mutant[a].

gene[b]	annotated function[b]	Fc[c]	*P*-value[d]	class[e]	classification details[e]
SCO2424	putative secreted protein	1.617	2.01E-08	c. e.	4.1.7 Gram positive exported/lipoprotein (439)
SCO2435	conserved hypothetical protein	0.526	0.00E+00	u. f.	0.0.2 Conserved in organism other than Escherichia coli (1322)
SCO2468	DNA primase	1.643	5.18E-09	m. m.	2.2.3 DNA - replication. repair. restr./modific'n (85)
SCO2480	putative secreted protein	1.918	0.00E+00	c. e.	4.1.7 Gram positive exported/lipoprotein (439)
SCO2520	putative membrane protein	0.526	0.00E+00	c. e.	4.1.6 Gram positive membrane (836)
SCO2525	hypothetical protein SCC121.28c	0.405	0.00E+00	u. f.	0.0.0 Unknown function. no known homologs (1049)
SCO2550	putative lipoprotein.	0.474	0.00E+00	c. e.	4.1.7 Gram positive exported/lipoprotein (439)
SCO2640	aspartate semialdehyde dehydrogenase	1.824	2.10E-13	s. m	3.1.13 Lysine (13)
SCO2691	hypothetical protein	2.234	0.00E+00	u. f.	0.0.0 Unknown function. no known homologs (1049)
SCO2751	conserved hypothetical protein SCC57A.22c.	2.009	0.00E+00	u. f.	0.0.2 Conserved in organism other than Escherichia coli (1322)
SCO2780	putative secreted protein	1.757	9.44E-12	c. e.	4.1.7 Gram positive exported/lipoprotein (439)
SCO2790	conserved hypothetical protein SCC105.21c	1.710	1.29E-10	u. f.	0.0.2 Conserved in organism other than Escherichia coli (1322)
SCO2791	hypothetical protein SCC105.22c	2.430	0.00E+00	u. f.	0.0.0 Unknown function. no known homologs (1049)
SCO2792	araC-family transcriptional regulator	0.383	0.00E+00	r.	6.3.2 AraC (33)
SCO2793	oligoribonuclease	1.966	0.00E+00	m. m.	2.1.2 Degradation of RNA (8)
SCO2797	putative cellobiose transport permease	1.722	6.51E-11	c. p.	1.5.0 Transport/binding proteins (520)
SCO2818	conserved hypothetical protein	1.613	2.55E-08	u. f.	0.0.2 Conserved in organism other than Escherichia coli (1322)
SCO2822	putative decarboxylase	0.424	0.00E+00	n. c.	7.0.0 Not classified (included putative assignments) (565)
SCO2823	putative decarboxylase	0.489	0.00E+00	n. c.	7.0.0 Not classified (included putative assignments) (565)
SCO2879	putative membrane protein	0.359	0.00E+00	c. e.	4.1.7 Gram positive exported/lipoprotein (439)
SCO2880	conserved hypothetical protein SCE6.17.	0.426	0.00E+00	u. f.	0.0.2 Conserved in organism other than Escherichia coli (1322)
SCO2881	conserved hypothetical protein SCE6.18	0.499	0.00E+00	u. f.	0.0.2 Conserved in organism other than Escherichia coli (1322)
SCO2883	putative cytochrome P450.	0.617	1.66E-08	s. m	3.5.3 Electron transport (71)
SCO2884	putative cytochrome P450.	0.624	4.40E-08	s. m	3.5.3 Electron transport (71)
SCO2911	conserved hypothetical protein	0.609	5.34E-09	u. f.	0.0.2 Conserved in organism other than Escherichia coli (1322)
SCO2912	hypothetical protein	0.551	3.00E-13	u. f.	0.0.0 Unknown function. no known homologs (1049)
SCO2919	hypothetical protein	0.408	0.00E+00	u. f.	0.0.0 Unknown function. no known homologs (1049)
SCO2921	putative membrane protein	0.196	0.00E+00	c. e.	4.1.6 Gram positive membrane (836)
SCO2927	putative 4-hydroxyphenylpyruvate dioxygenase	0.236	0.00E+00	s. m	3.4.2 Amino acids (28)
SCO2962	putative bi-functional transferase/deacetylase	1.746	1.73E-11	n. c.	7.0.0 Not classified (included putative assignments) (565)
SCO2987	putative regulatory protein	1.647	4.21E-09	r.	6.3.7 MarR (40)
SCO2994	putative tetR-family transcriptional regulator (fragment)	1.879	0.00E+00	r.	6.3.8 TetR (122)
SCO2995	putative ABC transporter integral membrane protein	2.315	0.00E+00	c. p.	1.5.0 Transport/binding proteins (520)
SCO2997	putative transferase	1.837	1.10E-13	m. m.	2.2.4 Glycoprotein (11)
SCO3061	putative integral membrane protein	1.632	9.45E-09	c. e.	4.1.6 Gram positive membrane (836)
SCO3089	putative ABC transporter ATP-binding protein	1.600	4.87E-08	c. p.	1.5.0 Transport/binding proteins (520)
SCO3123	ribose-phosphate pyrophosphokinase	1.891	0.00E+00	s. m	3.7.1 Purine ribonucleotide biosynthesis (21)
SCO3167	putative tetR-family transcriptional regulator	2.650	0.00E+00	r.	6.3.8 TetR (122)
SCO3177	putative membrane protein	1.618	1.90E-08	c. e.	4.1.6 Gram positive membrane (836)
SCO3197	putative 1-phosphofructokinase	1.958	0.00E+00	s. m	3.5.5 Glycolysis (25)

Additional file 2 – Complete set of genes differentially expressed in the *S. lividans adpA* mutant[a].

gene[b]	annotated function[b]	Fc[c]	*P*-value[d]	class[e]	classification details[e]
SCO3202	RNA polymerase principal sigma factor	2.499	0.00E+00	r.	6.2.1 sigma factor (66)
SCO3319	putative glutamyl-tRNA reductase	1.878	0.00E+00	s. m	3.2.6 Heme. porphyrin (16)
SCO3323	putative RNA polymerase sigma factor	0.389	0.00E+00	r.	6.2.1 sigma factor (66)
SCO3357	hypothetical protein	1.656	2.56E-09	u. f.	0.0.2 Conserved in organism other than Escherichia coli (1322)
SCO3421	conserved hypothetical protein	0.594	6.01E-10	u. f.	0.0.2 Conserved in organism other than Escherichia coli (1322)
SCO3495	putative aldolase	0.623	3.49E-08	s. m	3.4.3 Carbon compounds (91)
SCO3579	putative regulatory protein	0.310	0.00E+00	r.	6.5.0 Others (237)
SCO3752	putative ABC transporter ATP-binding protein	0.562	2.32E-12	c. p.	1.5.0 Transport/binding proteins (520)
SCO3767	conserved hypothetical protein	0.534	0.00E+00	u. f.	0.0.2 Conserved in organism other than Escherichia coli (1322)
SCO3768	putative translocase protein	0.601	1.57E-09	n. c.	7.0.0 Not classified (included putative assignments) (565)
SCO3776	putative membrane protein	1.722	1.75E-09	c. e.	4.1.6 Gram positive membrane (836)
SCO3810	putative gntR-family transcriptional regulator	1.762	6.98E-12	r.	6.3.3 GntR (51)
SCO3811	putative D-alanyl-D-alanine carboxypeptidase	1.628	1.12E-08	c. e.	4.1.9 Gram positive peptidoglycan. teichoic acid (40)
SCO3830	putative branched-chain alpha keto acid dehydrogenase E1 beta subunit	1.837	1.10E-13	s. m	3.5.7 Pyruvate dehydrogenase (10)
SCO3831	E1-alpha branched-chain alpha keto acid dehydrogenase	2.230	0.00E+00	s. m	3.5.7 Pyruvate dehydrogenase (10)
SCO3835	putative dehydrogenase	2.207	0.00E+00	n. c.	7.0.0 Not classified (included putative assignments) (565)
SCO3877	putative 6-phosphogluconate dehydrogenase	2.020	0.00E+00	s. m	3.5.6 Oxidative branch. pentose pwy (9)
SCO3917	conserved hypothetical protein	0.504	0.00E+00	u. f.	0.0.2 Conserved in organism other than Escherichia coli (1322)
SCO3945	putative cytochrome oxidase subunit I	3.386	0.00E+00	s. m	3.5.3 Electron transport (71)
SCO3946	putative cytochrome oxidase subunit II	3.594	0.00E+00	s. m	3.5.3 Electron transport (71)
SCO3947	putative ABC transporter	2.653	0.00E+00	c. p.	1.5.0 Transport/binding proteins (520)
SCO3965	conserved hypothetical protein	0.603	2.24E-09	u. f.	0.0.2 Conserved in organism other than Escherichia coli (1322)
SCO3966	putative secreted protein	0.549	2.10E-13	c. e.	4.1.7 Gram positive exported/lipoprotein (439)
SCO3967	conserved hypothetical membrane protein	0.406	0.00E+00	c. e.	4.1.6 Gram positive membrane (836)
SCO3968	putative integral membrane protein	0.443	0.00E+00	c. e.	4.1.6 Gram positive membrane (836)
SCO3971	conserved hypothetical protein	1.631	9.87E-09	u. f.	0.0.2 Conserved in organism other than Escherichia coli (1322)
SCO4000	hypothetical protein 2SC10A7.04c	1.877	0.00E+00	u. f.	0.0.0 Unknown function. no known homologs (1049)
SCO4032	putative marR regulatory protein	1.807	4.90E-13	r.	6.3.7 MarR (40)
SCO4034	putative RNA polymerase sigma factor	0.582	7.93E-11	r.	6.2.1 sigma factor (66)
SCO4049	putative antibiotic binding protein	1.708	1.40E-10	c. p.	1.4.2 Detoxification (90)
SCO4065	putative transposase	0.612	8.67E-09	e.	5.1.4 Transposon/insertion element-related functions (85)
SCO4113	conserved hypothetical protein	0.568	7.03E-12	u. f.	0.0.2 Conserved in organism other than Escherichia coli (1322)
SCO4114	sporulation associated protein	0.487	0.00E+00	c. p.	1.8.1 Differentiation/sporulation (11)
SCO4140	phosphate ABC transport system permease protein	0.584	1.14E-10	c. p.	1.5.0 Transport/binding proteins (520)
SCO4164	putative thiosulfate sulfurtransferase	0.483	0.00E+00	s. m	3.3.19 Sulfur metabolism (13)
SCO4165	conserved hypothetical protein	0.486	0.00E+00	u. f.	0.0.2 Conserved in organism other than Escherichia coli (1322)
SCO4187	putative membrane protein	0.245	0.00E+00	c. e.	4.1.6 Gram positive membrane (836)
SCO4215	putative GntR-family regulatory protein	1.964	0.00E+00	r.	6.3.3 GntR (51)
SCO4271	putative NADP-dependent alcohol dehydrogenase	1.795	1.05E-12	n. c.	7.0.0 Not classified (included putative assignments) (565)

Additional file 2 – Complete set of genes differentially expressed in the *S. lividans adpA* mutant[a].

gene[b]	annotated function[b]	Fc[c]	*P*-value[d]	class[e]	classification details[e]
SCO4275	putative histidine autokinase	1.730	4.26E-11	r.	6.1.1 Sensor kinase (85)
SCO4293	putative threonine synthase	0.362	0.00E+00	s. m	3.1.18 Threonine (4)
SCO4294	conserved hypothetical protein	0.431	0.00E+00	u. f.	0.0.2 Conserved in organism other than Escherichia coli (1322)
SCO4295	cold shock protein	0.217	0.00E+00	c. p.	1.6.1 Adaptations. atypical conditions (25)
SCO4296	chaperonin 2	0.566	5.11E-12	c. p.	1.3.1 Chaperones (15)
SCO4327	conserved hypothetical protein SCD12A.10c	1.662	1.88E-09	u. f.	0.0.2 Conserved in organism other than Escherichia coli (1322)
SCO4338	hypothetical protein SCD12A.21	0.544	1.10E-13	u. f.	0.0.2 Conserved in organism other than Escherichia coli (1322)
SCO4383	4-coumarate:CoA ligase	1.810	4.00E-13	m. m.	2.2.8 Polysaccharides - (cytoplasmic) (25)
SCO4393	conserved hypothetical protein	1.724	5.77E-11	u. f.	0.0.2 Conserved in organism other than Escherichia coli (1322)
SCO4427	conserved hypothetical protein	0.534	0.00E+00	u. f.	0.0.2 Conserved in organism other than Escherichia coli (1322)
SCO4428	putative secreted protein	0.550	3.00E-13	c. e.	4.1.7 Gram positive exported/lipoprotein (439)
SCO4498	putative proton transport protein	0.592	4.45E-10	c. p.	1.5.2 Cations (40)
SCO4513	hypothetical protein SCD35.20c	0.621	2.97E-08	u. f.	0.0.0 Unknown function. no known homologs (1049)
SCO4514	putative integral membrane protein	0.548	2.10E-13	c. e.	4.1.6 Gram positive membrane (836)
SCO4516	hypothetical protein SCD35.23c	0.374	0.00E+00	u. f.	0.0.0 Unknown function. no known homologs (1049)
SCO4521	putative secreted protein	0.610	1.78E-05	c. e.	4.1.7 Gram positive exported/lipoprotein (439)
SCO4671	putative lysR-family regulatory protein	1.734	9.99E-10	r.	6.3.6 LysR (38)
SCO4761	10 kD chaperonin cpn10	0.401	0.00E+00	c. p.	1.3.1 Chaperones (15)
SCO4762	60 kD chaperonin cpn60	0.440	0.00E+00	c. p.	1.3.1 Chaperones (15)
SCO4768	putative two-component regulator	0.586	1.44E-10	r.	6.3.11 LuxR (GerR) (23)
SCO4875	putative sugar transferase	0.600	1.42E-09	m. m.	2.2.8 Polysaccharides - (cytoplasmic) (25)
SCO4876	putative sugar translocase	0.586	1.44E-10	c. p.	1.5.5 Other (11)
SCO4880	putative transferase	0.597	9.05E-10	n. c.	7.0.0 Not classified (included putative assignments) (565)
SCO4881	putative polysaccharide biosynthesis related protein	0.558	1.24E-12	m. m.	2.2.8 Polysaccharides - (cytoplasmic) (25)
SCO4920	putative deoR-family transcriptional regulator	0.523	0.00E+00	r.	6.3.10 DeoR (13)
SCO4950	nitrate reductase gamma chain NarI3	0.211	0.00E+00	s. m	3.5.2 Anaerobic respiration (22)
SCO4951	putative aldoketoreductase	0.440	0.00E+00	s. m	3.8.1 PKS (102)
SCO4952	putative tetR-family transcriptional regulator	0.301	0.00E+00	r.	6.3.8 TetR (122)
SCO4974	putative deaminase	1.826	2.10E-13	n. c.	7.0.0 Not classified (included putative assignments) (565)
SCO5011	putative integral membrane protein	0.454	0.00E+00	c. e.	4.1.6 Gram positive membrane (836)
SCO5012	putative integral membrane protein	0.466	0.00E+00	c. e.	4.1.6 Gram positive membrane (836)
SCO5029	putative secreted protein	0.566	5.35E-12	c. e.	4.1.7 Gram positive exported/lipoprotein (439)
SCO5032	alkyl hydroperoxide reductase	0.576	2.95E-11	c. p.	1.4.2 Detoxification (90)
SCO5050	putative nucleotide-sugar dehydrogenase	1.852	0.00E+00	s. m	3.3.18 Sugar-nucleotide biosynthesis. conversions (11)
SCO5077	hypothetical protein	0.600	1.46E-09	s. m	3.8.1 PKS (102)
SCO5101	conserved hypothetical protein	0.584	1.11E-10	u. f.	0.0.2 Conserved in organism other than Escherichia coli (1322)
SCO5123	putative small membrane protein	0.343	0.00E+00	c. e.	4.1.6 Gram positive membrane (836)
SCO5240	hypothetical protein	2.246	0.00E+00	u. f.	0.0.2 Conserved in organism other than Escherichia coli (1322)
SCO5249	putative nucleotide-binding protein	0.551	3.00E-13	n. c.	7.0.0 Not classified (included putative assignments) (565)
SCO5390	putative alkanal monooxygenase (luciferase)	0.608	6.79E-08	n. c.	7.0.0 Not classified (included putative assignments) (565)

Additional file 2 – Complete set of genes differentially expressed in the *S. lividans adpA* mutant[a].

gene[b]	annotated function[b]	Fc[c]	*P*-value[d]	class[e]	classification details[e]
SCO5512	acetolactate synthase	1.672	1.06E-09	s. m	3.4.3 Carbon compounds (91)
SCO5521	hypothetical protein SC1C2.02	0.502	0.00E+00	u. f.	0.0.0 Unknown function. no known homologs (1049)
SCO5555	hypothetical protein SC1C2.36	2.828	0.00E+00	u. f.	0.0.0 Unknown function. no known homologs (1049)
SCO5556	histone-like DNA binding protein	2.388	0.00E+00	m. m.	2.2.2 Basic proteins - synthesis. modification (1)
SCO5650	putative membrane protein	0.578	4.33E-11	c. e.	4.1.6 Gram positive membrane (836)
SCO5741	putative membrane protein	2.502	0.00E+00	c. e.	4.1.6 Gram positive membrane (836)
SCO5742	putative membrane protein	2.094	0.00E+00	c. e.	4.1.6 Gram positive membrane (836)
SCO5751	putative membrane protein	0.613	9.87E-09	c. e.	4.1.6 Gram positive membrane (836)
SCO5789	hypothetical protein SC4H2.10c	2.001	0.00E+00	u. f.	0.0.0 Unknown function. no known homologs (1049)
SCO5790	hypothetical protein SC4H2.11c	1.704	1.81E-10	u. f.	0.0.0 Unknown function. no known homologs (1049)
SCO5793	diaminopimelate epimerase	1.784	1.97E-12	s. m	3.1.13 Lysine (13)
SCO5818	putative ABC transporter	1.666	1.52E-09	c. p.	1.5.0 Transport/binding proteins (520)
SCO5862	two-component regulator CutR	1.927	0.00E+00	r.	6.1.2 Response regulator (79)
SCO5863	two-component sensor (kinase)	1.714	1.04E-10	r.	6.1.1 Sensor kinase (85)
SCO6000	hypothetical protein	0.560	1.70E-12	u. f.	0.0.2 Conserved in organism other than Escherichia coli (1322)
SCO6004	putative secreted ATP/GTP binding protein	0.603	2.16E-09	c. e.	4.1.7 Gram positive exported/lipoprotein (439)
SCO6009	solute-binding protein	1.736	3.03E-11	c. p.	1.5.0 Transport/binding proteins (520)
SCO6010	probable ABC-transport system ATP binding protein	1.620	1.76E-08	c. p.	1.5.0 Transport/binding proteins (520)
SCO6019	conserved hypothetical protein SC1C3.07	1.727	3.78E-08	u. f.	0.0.2 Conserved in organism other than Escherichia coli (1322)
SCO6073	putative cyclase	0.466	0.00E+00	s. m	3.8.0 Secondary metabolism (165)
SCO6075	conserved hypothetical protein	1.892	0.00E+00	u. f.	0.0.2 Conserved in organism other than Escherichia coli (1322)
SCO6096	putative lipoprotein	0.557	9.60E-13	c. e.	4.1.7 Gram positive exported/lipoprotein (439)
SCO6097	sulfate adenylyltransferase subunit 1	0.579	4.60E-11	s. m	3.3.19 Sulfur metabolism (13)
SCO6098	sulfate adenylyltransferase subunit 2	0.519	0.00E+00	s. m	3.3.19 Sulfur metabolism (13)
SCO6099	adenylylsulfate kinase	0.544	1.10E-13	s. m	3.3.19 Sulfur metabolism (13)
SCO6100	phosphoadenosine phosphosulfate reductase	0.623	3.88E-08	s. m	3.3.19 Sulfur metabolism (13)
SCO6101	hypothetical protein SCBAC1A6.25c	0.476	0.00E+00	u. f.	0.0.0 Unknown function. no known homologs (1049)
SCO6102	putative nitrite/sulphite reductase	0.532	0.00E+00	s. m	3.5.2 Anaerobic respiration (22)
SCO6130	hypothetical protein SC9B2.17	0.410	0.00E+00	u. f.	0.0.2 Conserved in organism other than Escherichia coli (1322)
SCO6174	hypothetical protein SC6C5.10	0.538	0.00E+00	u. f.	0.0.2 Conserved in organism other than Escherichia coli (1322)
SCO6176	conserved hypothetical protein	0.459	0.00E+00	u. f.	0.0.2 Conserved in organism other than Escherichia coli (1322)
SCO6197	putative secreted protein	0.147	0.00E+00	c. e.	4.1.7 Gram positive exported/lipoprotein (439)
SCO6198	putative secreted protein	0.618	1.94E-08	c. e.	4.1.7 Gram positive exported/lipoprotein (439)
SCO6227	hypothetical protein SC2H4.09	1.713	2.68E-09	u. f.	0.0.0 Unknown function. no known homologs (1049)
SCO6230	putative sugar transport system permease protein	1.626	1.24E-08	c. p.	1.5.0 Transport/binding proteins (520)
SCO6306	putative oxidoreductase	1.604	4.50E-07	n. c.	7.0.0 Not classified (included putative assignments) (565)
SCO6373	putative integral membrane protein	0.585	4.21E-05	c. e.	4.1.6 Gram positive membrane (836)
SCO6376	putative integral membrane protein	0.603	3.94E-08	c. e.	4.1.6 Gram positive membrane (836)
SCO6384	putative integral membrane lysyl-tRNA synthetase	0.590	1.39E-07	m. m.	2.2.1 Amino acyl tRNA syn; tRNA modific'n (34)
SCO6458	conserved hypothetical protein SC9B5.25	1.636	7.37E-09	u. f.	0.0.2 Conserved in organism other than Escherichia coli (1322)

Additional file 2 – Complete set of genes differentially expressed in the *S. lividans adpA* mutant[a].

gene[b]	annotated function[b]	Fc[c]	*P*-value[d]	class[e]	classification details[e]
SCO6482	conserved hypothetical protein	0.538	0.00E+00	u. f.	0.0.2 Conserved in organism other than Escherichia coli (1322)
SCO6507	putative gas vesicle synthesis protein	0.410	0.00E+00	c. p.	1.6.1 Adaptations. atypical conditions (25)
SCO6509	hydrophobic protein	0.398	0.00E+00	n. c.	7.0.0 Not classified (included putative assignments) (565)
SCO6510	conserved hypothetical protein SC1E6.19c	0.406	0.00E+00	u. f.	0.0.2 Conserved in organism other than Escherichia coli (1322)
SCO6645	putative transport system permease protein	1.864	0.00E+00	c. p.	1.5.0 Transport/binding proteins (520)
SCO6659	glucose-6-phosphate isomerase	2.018	0.00E+00	s. m	3.5.5 Glycolysis (25)
SCO6660	hypothetical protein SC5A7.10c	2.207	0.00E+00	u. f.	0.0.2 Conserved in organism other than Escherichia coli (1322)
SCO6661	glucose-6-phosphate 1-dehydrogenase	2.614	0.00E+00	s. m	3.5.6 Oxidative branch. pentose pwy (9)
SCO6662	transaldolase	2.180	0.00E+00	s. m	3.3.9 Non-oxidative branch. pentose pwy (8)
SCO6685	putative two-component system response regulator	0.624	3.94E-08	r.	6.1.2 Response regulator (79)
SCO6691	putative phospholipase C	0.414	0.00E+00	s. m	3.4.4 Fatty acids (59)
SCO6799	threonine 3-dehydrogenase.	1.638	6.68E-09	s. m	3.4.2 Amino acids (28)
SCO6808	putative ArsR-family transcriptional regulator	0.268	0.00E+00	r.	6.3.13 ArsR (15)
SCO6818	putative phosphoglycerate mutase.	0.389	0.00E+00	s. m	3.5.5 Glycolysis (25)
SCO6820	putative oxidoreductase.	0.338	0.00E+00	n. c.	7.0.0 Not classified (included putative assignments) (565)
SCO6824	putative phosphonopyruvate decarboxylase (fragment).	0.564	3.48E-12	n. c.	7.0.0 Not classified (included putative assignments) (565)
SCO6830	putative ArsR-family transcriptional regulator	0.284	0.00E+00	r.	6.3.13 ArsR (15)
SCO6831	hypothetical protein SC4A9.08	0.602	3.14E-08	u. f.	0.0.0 Unknown function. no known homologs (1049)
SCO6903	hypothetical protein SC1B2.09	0.617	2.03E-07	u. f.	0.0.2 Conserved in organism other than Escherichia coli (1322)
SCO6926	hypothetical protein SC1B2.32.	0.600	4.20E-07	u. f.	0.0.2 Conserved in organism other than Escherichia coli (1322)
SCO6937	putative DNA-binding protein	0.545	1.10E-13	r.	6.5.0 Others (237)
SCO6939	conserved hypothetical protein SC1G8.11c.	0.485	0.00E+00	u. f.	0.0.2 Conserved in organism other than Escherichia coli (1322)
SCO6979	probable solute-binding lipoprotein.	0.611	7.37E-09	c. p.	1.5.0 Transport/binding proteins (520)
SCO7011	putative membrane transport protein.	1.764	6.21E-12	c. p.	1.5.0 Transport/binding proteins (520)
SCO7070	hypothetical protein SC4G1.36	1.649	8.68E-07	u. f.	0.0.0 Unknown function. no known homologs (1049)
SCO7221	putative polyketide synthase.	1.692	3.42E-10	s. m	3.8.1 PKS (102)
SCO7233	putative secreted protein.	0.309	0.00E+00	c. e.	4.1.7 Gram positive exported/lipoprotein (439)
SCO7251	conserved hypothetical protein	0.574	2.21E-11	u. f.	0.0.2 Conserved in organism other than Escherichia coli (1322)
SCO7344	putative secreted protein	0.467	0.00E+00	c. e.	4.1.7 Gram positive exported/lipoprotein (439)
SCO7398	putative membrane transport protein.	1.678	1.52E-08	c. p.	1.5.0 Transport/binding proteins (520)
SCO7399	possible binding-protein-dependent transport lipoprotein.	1.807	4.90E-13	c. p.	1.5.0 Transport/binding proteins (520)
SCO7400	putative ABC-transport protein. ATP-binding component.	1.772	3.90E-12	c. p.	1.5.0 Transport/binding proteins (520)
SCO7409	putative binding-protein dependent transport protein.	2.072	0.00E+00	c. p.	1.5.0 Transport/binding proteins (520)
SCO7410	putative binding-protein dependent transport protein.	1.707	1.47E-10	c. p.	1.5.0 Transport/binding proteins (520)
SCO7449	putative membrane protein.	1.634	8.47E-09	c. e.	4.1.6 Gram positive membrane (836)
SCO7472	putative phenylacetic acid degradation protein PaaB	1.811	4.00E-13	s. m	3.4.3 Carbon compounds (91)
SCO7473	putative phenylacetic acid degradation protein PaaC	1.716	9.44E-11	s. m	3.4.3 Carbon compounds (91)
SCO7474	putative phenylacetic acid degradation protein PaaD	2.033	0.00E+00	s. m	3.4.3 Carbon compounds (91)
SCO7475	putative phenylacetic acid degradation NADH oxidoreductase PaaE	1.737	2.93E-11	s. m	3.4.3 Carbon compounds (91)

Additional file 2 – Complete set of genes differentially expressed in the *S. lividans adpA* mutant[a].

gene[b]	annotated function[b]	Fc[c]	*P*-value[d]	class[e]	classification details[e]
SCO7477	putative membrane protein	1.657	2.44E-09	c. e.	4.1.7 Gram positive exported/lipoprotein (439)
SCO7549	hypothetical protein	0.447	0.00E+00	u. f.	0.0.2 Conserved in organism other than Escherichia coli (1322)
SCO7550	putative secreted hydrolase	0.334	0.00E+00	c. e.	4.1.7 Gram positive exported/lipoprotein (439)
SCO7603	putative LysR-family transcriptional regulator	1.600	4.70E-08	r.	6.3.6 LysR (38)
SCO7631	putative secreted protein	0.355	0.00E+00	c. e.	4.1.7 Gram positive exported/lipoprotein (439)
SCO7648	putative two-component system response regulator	0.616	1.48E-08	r.	6.1.2 Response regulator (79)
SCO7657	putative secreted protein	0.033	0.00E+00	c. e.	4.1.7 Gram positive exported/lipoprotein (439)
SCO7658	hypothetical protein SC10F4.31	0.103	0.00E+00	u. f.	0.0.0 Unknown function. no known homologs (1049)
SCO7659	putative oxidoreductase	0.511	0.00E+00	n. c.	7.0.0 Not classified (included putative assignments) (565)
SCO7685	conserved hypothetical protein	1.632	9.11E-09	s. m	3.8.0 Secondary metabolism (165)
SCO7697	putative secreted hydrolase	0.557	9.60E-13	n. c.	7.0.0 Not classified (included putative assignments) (565)
SCO7700	putative cyclase	1.636	1.49E-06	n. c.	7.0.0 Not classified (included putative assignments) (565)
SCO7714	putative acetyltransferase	0.516	0.00E+00	n. c.	7.0.0 Not classified (included putative assignments) (565)
SCO7774	putative secreted protein	0.589	2.62E-10	c. e.	4.1.7 Gram positive exported/lipoprotein (439)

a. Genes expression in the *S. lividans adpA* mutant was compared to that in the wild-type, using *S. coelicolor* microarrays. The complete data set is presented in this file. A selected subset of the genes extracted from Additional file 2 is shown in Table 1.

b. Gene names and annotated function are from the StrepDB database [7].

c. Fold change (Fc) in gene expression in the *S. lividans adpA* mutant with respect to the wild-type strain 1326.

d. From a Student's *t*-test applying the Benjamini and Hochberg multiple testing correction (*P*-value <0.05).

e. From the protein classification scheme for the *S. coelicolor* genome available on the Welcome Trust Sanger Institute database [37]: unknown function (u. f.), cell process (c. p.), macromolecule metabolism (m. m.), small molecule metabolism (s. m.), cell envelope (c. e.), extrachromosomal (e.), regulation (r.) and not classified (n. c).
