# Supplementary material for: Identified members of the Streptomyces lividans AdpA regulon involved in differentiation and secondary metabolism
Source: BMC Microbiol. 2014 Apr 3;14:81. doi: 10.1186/1471-2180-14-81 (PMC4021200; doi:10.1186/1471-2180-14-81)
Supplement: Additional file 3: Figure S1 — Effect of the mutation of one AdpA-binding site in the S. lividans hyaS promoter on AdpA-binding specificity. Mutation of an AdpA-binding site in the S. lividans hyaS promoter region prevents formation of an AdpA-DNA complex in vitro. Sequence of the mutated AdpA-binding site (at -129 nt) and EMSA performed with the mutated hyaS promoter region are shown. [file 1471-2180-14-81-S3.pdf]

a

AdpA-binding site

***hyaS* WT** GATCCGGCCA
CTAGGCCGGT

*Eco*RI

***hyaS* Mut** GATC**GAATTC**
CTAG**CTTGGC**

b

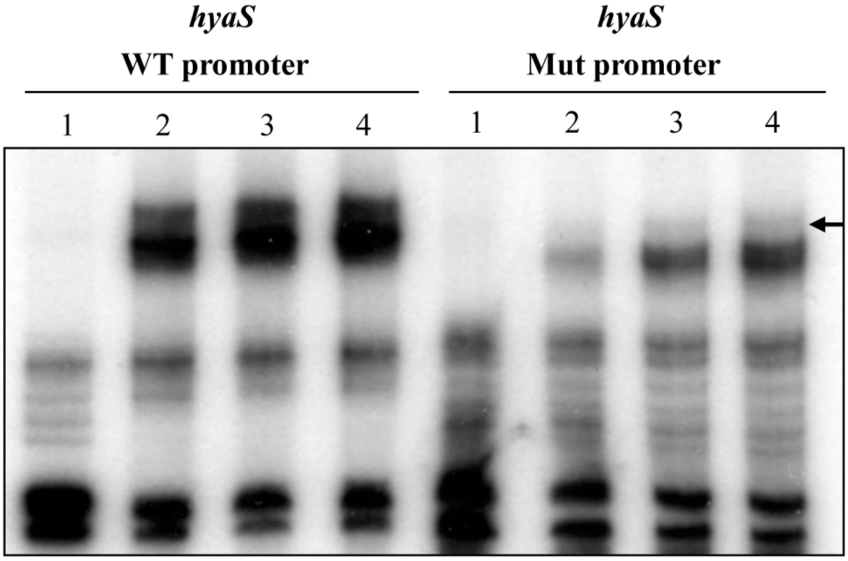

Additional file 3 – Effect of the mutation of one AdpA-binding site in the *S. lividans hyaS* promoter on AdpA-binding specificity.

a. One of the putative *S. lividans* AdpA-binding sites (position -129nt from translation start site) was mutated by PCR. A PCR was performed using the oligonucleotide pairs GShyaS-XbaI (5'-CGGTCTAGAACTGCCGCTCAGCCGTCA-3') and GShyaS-mutrev (5'-GGCCGGAGGT**GAATTC**GATCGCAGCACTTTC-3') and generated amplicon A (128 bp). The amplicon B (120 bp) was obtained with the oligonucleotides GShyaS-mutfor (5'-GAAAGTGCTGCGATC**GAATTC**ACCTCCGGCCAT-3') and GShyaS-BamHI (5'-CGGGGATCCGAAGGAAGCAGGCGGGCACT-3'). Since amplicons A and B had a 31 nucleotides sequence shared, a fusion PCR (amplicon C) was performed using amplicons A

and B as matrices DNA and GShyaS-BamHI/GShyaS-XbaI as oligonucleotides. Presence of the mutated AdpA site was checked by digestion of amplicon C by *Eco*RI. The amplicon C was digested by *Xba*I and *Bam*HI and cloned in pUC18. Mutation of the AdpA binding site was confirmed by DNA sequencing. A wild type PCR was also performed on chromosomal DNA using GShyaS-BamHI/GShyaS-XbaI oligonucleotides (amplicon D).

b. Two radiolabeled PCR were performed with GShyaS-1/GShyaS-2 oligonucleotides (Table S1) using amplicons C or D as DNA matrices allowing amplification of the Mut or WT promoters (201 bp). These DNA fragment were mixed with 0 (lane 1), 5.7 (lane 2), 11.4 (lane 3) or 17.1 (lane 4) pmoles of purified AdpA-His$_6$ and EMSA was performed as described in Figure 2. Arrow indicates position where a specific protein-DNA complex is absent. In this figure, a difference profile of the radiolabelled probe appeared as a consequence of using a Phusion High Fidelity Polymerase (buffer GC, Biolabs) for the PCR instead of the FlexiGoTaq polymerase (Promega) in Figure 2.
