# Supplementary material for: Identified members of the Streptomyces lividans AdpA regulon involved in differentiation and secondary metabolism
Source: BMC Microbiol. 2014 Apr 3;14:81. doi: 10.1186/1471-2180-14-81 (PMC4021200; doi:10.1186/1471-2180-14-81)
Supplement: Additional file 4: Table S3 — Comparison of gene expression profiles between S. coelicolor bldA-dependent and S. lividans AdpA-dependent genes. Comparison of the gene expression profiles of some S. coelicolor bldA-dependent genes whose S. lividans orthologs are AdpA-dependent (see Additional file 2: Table S2). Putative AdpA-binding sites were identified in silico (see Additional file 5: Table S4), suggesting that in the S. coelicolor bldA mutant, the adpA translation defect leads to bldA-dependence of the genes identified previously [42,47,48]. [file 1471-2180-14-81-S4.pdf]

Additional file 4 – Comparison of gene expression profiles between *S. coelicolor bldA*-dependent and *S. lividans* AdpA-dependent genes

**genes**[a]	**genes**[b]	**annotated function**[b]	**in *adpA* *S. lividans***[c]	**in *bldA* *S. coelicolor***[d]	**AdpA-binding site**[e]
TTA-containing genes:					
SCO0383	SLI0341	hypothetical protein	down		no
SCO0399	SLI0357	putative membrane protein	down		no
SCO2792 *adpA*	SLI3139	araC-family transcriptional regulator	down		putative
SCO3776	SLI4019	putative membrane protein	up		no
SCO4114	SLI4345	sporulation associated protein	down		putative
SCO4671	SLI4944	putative lysR-family regulatory protein	up		putative
SCO6075	SLI6468	conserved hypothetical protein	up		no
SCO6384	-	putative integral membrane lysyl-tRNA synthetase	down		putative
SCO7070	SLI7275	hypothetical protein SC4G1.36	up		putative
SCO7233	SLI7449	putative secreted protein.	down		no
SCO7251	SLI7467	conserved hypothetical protein	down		putative
Non TTA-containing gene:					
[illegible]	[illegible]	[illegible]	[illegible]	[illegible]	[illegible]
SCO0392	SLI0350	putative methyltransferase	down	down	no
SCO0498 *cchB*	**SLI0458***	putative peptide monooxygenase	down	down	yes
SCO0499 *cchA*	**SLI0459***	putative formyltransferase	down	down	yes
SCO0762 *sti1*	**SLI0743***	protease inhibitor precursor	down	down	yes
SCO1222	SLI1501	conserved hypothetical protein	down	down	putative
SCO1626 *rarE*	SLI1930	putative cytochrome P450	down	down	no
SCO1968 *glpQ2*	SLI2284	putative secreted hydrolase	down	down	putative
SCO2435	SLI2769	conserved hypothetical protein	down	down	putative
SCO2780 *desE*	SLI3127	putative secreted protein	up	down	no
SCO3123	SLI3480	ribose-phosphate pyrophosphokinase	up	up	putative
SCO3917	SLI4175	conserved hypothetical protein	down	down	putative
SCO4187	SLI4427	putative membrane protein	down	down	putative
SCO4295 *scoF4*	SLI4532	cold shock protein	down	down	putative
SCO4762 *groEL1*	SLI5032	60 kD chaperonin cpn60	down	down	putative
SCO5123	SLI5404	putative small membrane protein	down	down	putative
SCO5249	SLI5540	putative nucleotide-binding protein	down	down	putative
SCO6197	**SLI6586***	putative secreted protein	down	down	yes
SCO6198	**SLI6587**	putative secreted protein	down	down	yes
SCO6808	SLI1107	putative ArsR-family transcriptional regulator	down	down	putative
SCO7221	SLI7437	putative polyketide synthase.	up	up	putative
SCO7399 *cdtB*	SLI7618	possible binding-protein-dependent transport lipoprotein	up	up	no
SCO7400 *cdtC*	SLI7619	putative ABC-transport protein. ATP-binding component	down	down	no
SCO7657 *hyaS*	**SLI7885***	putative secreted protein	down	down	yes

a. *bldA*-dependent TTA-containing genes according to Li et al. [46]. SCO6384 might not have an ortholog gene in *S. lividans*.

b. Gene names and annotated function are from the StrepDB database [7]. *S. lividans* genes indicated with a star were analyzed by qRT-PCR and genes in bold were analyzed by EMSA experiments for direct *in vitro* AdpA binding (this study, [25]).

c. Gene expression in the *S. lividans adpA* mutant determined from expression profiles (Additional file 2).

d. Gene expression and/or protein levels in the *S. coelicolor bldA* mutant [42, 47, 48].

e. Putative *S. coelicolor* AdpA-binding sites identified *in silico* using PREDetector [39] (score values are indicated in Additional file 5). We confirmed that *S. lividans* AdpA binds to some promoter by EMSA experiments in Figure 2 [25].
