# Supplementary material for: Identified members of the Streptomyces lividans AdpA regulon involved in differentiation and secondary metabolism
Source: BMC Microbiol. 2014 Apr 3;14:81. doi: 10.1186/1471-2180-14-81 (PMC4021200; doi:10.1186/1471-2180-14-81)
Supplement: Additional file 5: Table S4 — Putative S. coelicolor AdpA-binding sites upstream from the S. lividans AdpA-dependent genes. We identified putative AdpA-binding sites in silico using the S. coelicolor genome and we analysed orthologs of S. lividans AdpA-dependent genes (based on our microarray data); the sequences and positions of the sites with the highest scores according to PREDetector are shown. S. coelicolor, S. lividans and S. griseus ortholog genes are indicated and previously identified direct or probably direct S. griseus AdpA-dependent genes are highlighted. [file 1471-2180-14-81-S5.pdf]

Additional file 5 – Putative *S. coelicolor* AdpA-binding sites upstream from the *S. lividans* AdpA-dependent genes[a].

gene[b]	function[c]	cis-element[c]	score[c]	position[c]	gene[d]	Fc[e]	gene[b]	gene name[f]	Co-transcript gene[g]
SCO0169	hypothetical protein	tgacgtgatg	3.64	-7	SLI0098	0.621	-		-
SCO0171	nicotinate phosphoribosyltransferase	cggcgggaac	6.11	-119	SLI0101	0.615	-		-
SCO0197	hypothetical protein	cggcgagttc	4.39	-85	SLI0139	1.82	-		-
SCO0200	hypothetical protein	attccggcca	8.99	-9	SLI0142	1.656	-		SCO0199/**SCO0198**
SCO0216	nitrate reductase subunit alpha NarG2	gttctggccc	5.0	-10	SLI0158	1.745	-		**SCO0217**/SCO0218 /**SCO0219**
SCO0229	short chain dehydrogenase	aaaatcgaca	3.1	-72	SLI0172	1.988	-		**SCO0230**
SCO0231	hypothetical protein	ctaccggccc	4.07	-97	SLI0175	0.122	SGR4343		
SCO0268	hypothetical protein	tggctcgttt	6.41	-114	SLI0220	0.1	-		-
SCO0379	catalase	gtgccggaac	3.87	-173	SLI0337	0.483	-	*katA*	-
SCO0382	UDP-glucose/GDP-mannose dehydrogenase	gaacgggtca	4.52	-9	SLI0340	0.491	-		-
SCO0384	hypothetical protein	gggcgaattc	3.13	-45	SLI0342	0.611	-		**SCO0385/SCO0386**
SCO0402*	hypothetical protein	attcgggcca	7.63	-151	SLI0360	0.578	-		-
SCO0453	solute-binding lipoprotein	ggtcccgcca	5.89	-84	SLI0411	1.876	-		-
SCO0494	iron-siderophore binding lipoprotein	tgtcgcgcca	4.36	-28	SLI0454	0.615	SGR6714	*cchF*	**SCO0493**
SCO0498	peptide monooxygenase	attcgagcca	6.91	-91	**SLI0458**	0.336	SGR6710	*cchB*	SCO0497/SCO0496/ **SCO0495**
SCO0499	formyltransferase	attcgagcca	6.91	-95	**SLI0459**	0.374	SGR6709	*cchA*	-
SCO0561	Fe regulatory protein	tggcgggcac	4.44	-84	SLI0523	0.613	-	*furS. furA*	SCO0560
SCO0592	hypothetical protein	gggcgcgtac	3.41	-105	SLI0556	0.624	-		-
SCO0643	cellulose-binding protein	gaatgagcca	5.28	-134	SLI0614	2.002	-		-
SCO0682	hypothetical protein	tttccggacg	5.03	-21	SLI0655	0.375	SGR4457		
SCO0705	hypothetical protein	gggccggatc	6.53	-94	SLI0681	0.579	SGR92t SGR7047t		-
**SCO0762**	protease inhibitor protein	tggcgtgatc	7.19	-235	**SLI0743**	0.124	**SGR551**	*sti1 sgiA*	-
SCO0771	hypothetical protein	tggctgaacc	5.08	-10	SLI0752	0.612	-		**SCO0770**
SCO0774*	cytochrome P450	tggcgcgatc	6.9	-180	**SLI0755**	0.075	SGR264		SCO0773
SCO0775*	hypothetical protein	tggcgcgatc	6.9	-140	**SLI0756**	0.424	SGR1346		-
SCO0827	hypothetical protein	tgtctgctta	3.4	-68	SLI0811	0.516	-		-
SCO0863	hypothetical protein	gttccggccg	6.75	-141	SLI0848	0.225	-		SCO0862
SCO0864	ECF family RNA polymerase sigma factor	agtcccgccg	4.45	-94	SLI0849	0.574	SGR6228		-

Additional file 5 – Putative *S. coelicolor* AdpA-binding sites upstream from the *S. lividans* AdpA-dependent genes[a].

gene[b]	function[c]	cis-element[c]	score[c]	position[c]	gene[d]	Fc[e]	gene[b]	gene name[f]	Co-transcript gene[g]
SCO0865	hypothetical protein	catcctgaca	3.27	-18	SLI0850	0.621	-		-
SCO0919	hypothetical protein	tgtccgcatc	5.1	-48	SLI1149	0.621	SGR6643		-
SCO0929	hypothetical protein	tggccggacg	5.19	-201	SLI1160	0.419	SGR710		SCO0928
SCO1032*	putative ABC transport system ATP-binding	cggccgaact	4.36	#	SLI1304	2.138	-		**SCO1033**/SCO1032 /**SCO1031**
SCO1134*	oxidoreductase. iron-sulfur binding subunit	gattcggcca	8.17	-169	SLI1410	0.584	SGR6436		**SCO1133**/SCO1132 /**SCO1131**
SCO1179	hypothetical protein	gtacgggccc	4.05	-37	SLI1456	1.649	SGR3185		**SCO1178**
SCO1183	hypothetical protein	cgaccggaag	3.2	-18	SLI1460	1.682	SGR0892		SCO1184/**SCO1185**
SCO1186	LacI family transcriptional regulator	gtatcggacg	3.66	-35	SLI1464	1.657	-		-
SCO1196	hypothetical protein	tggccggacc	6.53	-95	SLI1475	0.493	SGR493		-
SCO1222	hypothetical protein	ctgcgggaac	3.76	-103	SLI1501	0.509	SGR5233		SCO1223
SCO1379	hypothetical protein	agtccggccc	4.56	-24	detected	1.8	SGR6150		-
SCO1407	hypothetical protein	tggcggactc	4.02	-10	SLI1704	0.617	SGR6124		-
SCO1430	TetR family transcriptional regulator	gggcgggacc	3.52	-34	SLI1728	1.685	-		-
SCO1444	chitinase	catccggact	3.65	-320	SLI1742	1.984	SGR6088		-
SCO1565	glycerophosphoryl diester phosphodiesterase	cggccggaac	6.75	-82	SLI1868	0.531	**SGR5973**	*glpQ1*	-
SCO1593	transcriptional regulator	attcacgccg	5.44	-193	SLI1845	1.86	SGR5994		-
SCO1630	hypothetical protein	tgtcgggatc	6.71	-74	SLI1934	0.505	SGR1063	*cvn9. rarA*	SCO1629/SCO1628
SCO1674	hypothetical protein	cggcggaatc	5.69	-154	SLI1979	0.564	SGR5829	*chpC*	-
SCO1684	hypothetical protein	gaatgcgcca	5.36	-161	SLI1989	1.626	SGR5819		-
SCO1715	homogentisate 1.2-dioxygenase	gttccggtca	6.38	-160	SLI2018	0.601	SGR5788	*hgd*	**SCO1716/SCO1717**
SCO1776*	CTP synthetase	cttccggcca	7.25	-170	SLI2080	1.744	SGR5721	*pyrG*	-
SCO1800	hypothetical protein	cggccggacc	4.69	-65	SLI2108	0.256	SGR5696	*chpE*	-
SCO1821	molybdenum cofactor biosynthesis protein A	cggcccgaac	5.39	-61	SLI2130	1.679	SGR5674	*moaA*	-
SCO1864	acetyltransferase	atttcggaca	6.71	-203	SLI2175	2.903	SGR5635	*ectA*	-
SCO1865	diaminobutyrate--2-oxoglutarate aminotransferase	cggccgggac	3.24	-78	SLI2176	3.154	SGR5634	*ectB*	-
SCO1867	hydroxylase	gaagtggcca	4.62	-3	SLI2178	3.029	SGR5632	*ectD*	-
SCO1887	integral membrane transport protein	gttcgggtcg	3.18	-95	SLI2198	1.656	SGR210		**SCO1886/SCO1885 /SCO1884/SCO188 3**
SCO1968	hydrolase	cattcagcct	3.75	-92	SLI2284	0.537	SGR5556	*glpQ2*	SCO1969

Additional file 5 – Putative *S. coelicolor* AdpA-binding sites upstream from the *S. lividans* AdpA-dependent genes[a].

gene[b]	function[c]	cis-element[c]	score[c]	position[c]	gene[d]	Fc[e]	gene[b]	gene name[f]	Co-transcript gene[g]
SCO2068*	alkaline phosphatase	tggccggttc	8.09	-91	SLI2391	0.621	-		-
SCO2174	transferase	gggccggtac	5.41	-94	SLI2501	1.819	-		-
SCO2183	2-oxoacid dehydrogenase subunit E1	gttacagtca	3.68	-115	SLI2510	1.647	SGR5327	*aceE1*	-
SCO2212*	hypothetical protein	gttccggcca	8.59	-72	detected	0.516	-		-
SCO2396	hypothetical protein	cggcggattt	5.28	-57	SLI2728	1.734	SGR5099		-
SCO2424	hypothetical protein	cggccggtac	5.94	-113	SLI2756	1.617	SGR1042		**SCO2425**
SCO2435	hypothetical protein	gatcgggccg	5.7	-156	SLI2769	0.526	-		**SCO2436**
SCO2525	hypothetical protein	aatttggaca	5.8	-14	SLI2861	0.405	-		-
SCO2550	lipoprotein	catacggcca	5.58	-52	SLI2886	0.474	-		-
SCO2640	aspartate-semialdehyde dehydrogenase	gggcgggaag	4.24	-166	SLI2981	1.824	SGR4887	*asd1*	-
SCO2790	hypothetical protein	tgtccgggaa	3.36	-65	SLI3137	1.71	SGR4743		-
SCO2791*	hypothetical protein	gaaccggcca	8.09	-234	SLI3138	2.43	-		-
**SCO2792***	AraC family transcription regulator	gaaccggcca	8.09	-148	SLI3139	0.383	**SGR4742**	*adpA bldH*	-
SCO2793	oligoribonuclease	aattcagccc	5.48	-133	SLI3140	1.966	SGR4741	*ornA*	-
SCO2797	cellobiose transport permease	gaaccggccg	6.25	-9	SLI3145	1.722	-		-
SCO2818	hypothetical protein	cttccagccg	4.69	-200	SLI3166	1.613	SGR4718		-
SCO2823	decarboxylase	agacaggccg	3.21	-185	SLI3172	0.489	-		SCO2822
SCO2879	hypothetical protein	ttttcggcca	7.69	-448	SLI3224	0.359	-	*cvnA12*	SCO2880/SCO2881
SCO2919	hypothetical protein	cgacccgcca	3.74	-105	SLI3263	0.408	SGR4620		-
SCO2921*	hypothetical protein	tttgcggaca	4.62	-260	detected	0.196	**SGR4618**	*adbS3-orfa*	-
SCO2987	regulatory protein	gagcacgcca	3.68	-79	SLI3331	1.647	SGR6918	*ohrR*	-
SCO2997	transferase	ttgcccgatt	5.59	-83	SLI3341	1.837	-		**SCO2998**
SCO3061	hypothetical protein	attgacgcca	5.03	-36	SLI3411	1.632	SGR4476		-
SCO3123	ribose-phosphate pyrophosphokinase	tgaccggaaa	6.21	#	SLI3480	1.891	SGR4383	*prsA2*	-
SCO3167	TetR family transcriptional regulator	cgaccggaac	4.54	-48	SLI3521	2.65	SGR4317		-
SCO3177*	hypothetical protein	tggctgatat	6.73	-75	SLI3531	1.618	SGR4301		-
SCO3202	RNA polymerase principal sigma factor	aatccggaca	7.75	-145	SLI3556	2.499	SGR4276	*hrdD*	-
**SCO3323***	RNA polymerase sigma factor	gttccggtca	6.38	-469	SLI3667	0.389	**SGR4151**	*bldN. adsA*	-
SCO3357	hypothetical protein	cggcgagtac	4.08	-14	SLI3699	1.656	SGR4124	*cseA*	-
SCO3495	aldolase	tgtcctgttc	5.47	-21	**-**	0.623	-		**SCO3494**
**SCO3579***	regulatory protein	tggcccgaac	7.23	-135	SLI3822	0.31	SGR3340	*wblA*	-

Additional file 5 – Putative *S. coelicolor* AdpA-binding sites upstream from the *S. lividans* AdpA-dependent genes[a].

gene[b]	function[c]	cis-element[c]	score[c]	position[c]	gene[d]	Fc[e]	gene[b]	gene name[f]	Co-transcript gene[g]
SCO3767	hypothetical protein	cttcccgtca	4.4	-50	SLI4011	0.534	SGR3815		-
SCO3768	translocase	catcgggccc	3.83	-99	SLI4012	0.601	-		-
SCO3810	GntR family transcriptional regulator	tatccggacg	5.34	-57	SLI4061	1.762	SGR3772		**SCO3809**
SCO3811	D-alanyl-D-alanine carboxypeptidase	tatccggacg	5.34	-175	SLI4062	1.628	SGR3768	*dacA*	-
SCO3831	E1-alpha branched-chain alpha keto acid dehydrogenase	tggctacatg	3.37	-98	SLI4083	2.23	SGR3748	*bkdA2*	SCO3830/**SCO3829**
SCO3835	dehydrogenase	tgtcccgacc	3.62	-109	SLI4087	2.207	SGR3745		-
SCO3877	6-phosphogluconate dehydrogenase	gaacccgccg	5.61	-55	SLI4134	2.02	SGR3702		**SCO3876/SCO3875**
SCO3917*	hypothetical protein	ctttcggcca	6.52	-72	SLI4175	0.504	SGR3663		-
SCO3945	cytochrome oxidase subunit I	tgtcccgatt	6.39	-88	SLI4193	3.386	SGR3646	*cydA*	SCO3946
SCO3947	ABC transporter	catcccgccg	5.08	-30	SLI4195	2.653	SGR3644	*cydCD*	-
SCO3968*	hypothetical protein	tggcgggctc	4.75	-10	SLI4216	0.443	SGR3623		-
SCO3971	hypothetical protein	tggccggtac	7.78	-465	SLI4220	1.631	SGR3620		-
SCO4032	marR regulatory protein	aaaagggccg	3.31	-73	SLI4266	1.807	SGR3555		**SCO4031**
SCO4034	RNA polymerase sigma factor	tgtcggcaac	4.15	-128	SLI4268	0.582	SGR3552	*sigN*	-
SCO4049*	antibiotic binding protein	tattcggcca	8.0	-62	SLI4283	1.708	SGR4229		**SCO4050/SCO4051/SCO4052**
SCO4113	hydroxyglutarate oxidase	aaacccgtca	5.64	-52	SLI4344	0.568	SGR3901		-
SCO4114*	sporulation associated protein	tggcgggatt	8.66	-117	SLI4345	0.487	SGR3902		-
SCO4164	thiosulfate sulfurtransferase	gttgccgcca	5.7	-170	SLI4405	0.483	SGR3965	*cysA*	SCO4165
SCO4187*	hypothetical protein	tggcgagaag	5.39	-145	SLI4427	0.245	-		-
SCO4215	GntR family transcriptional regulator	gatgaggccg	3.74	-294	SLI4452	1.964	-	*xlnR*	-
SCO4293	threonine synthase	cggcccgatg	4.36	-145	SLI4530	0.362	SGR3228		SCO4294
SCO4295*	cold shock protein	attctcgcca	7.13	-193	SLI4532	0.217	SGR3226	*scoF4*	-
SCO4296	chaperonin GroEL	cggcccgatc	5.7	-210	SLI4533	0.566	SGR3225	*groEL2*	-
SCO4327	hypothetical protein	cgtcccgttc	3.34	-46	SLI4563	1.662	SGR3174		**SCO4326**
SCO4383	4-coumarate:CoA ligase	gatcaggccg	5.99	-122	SLI4617	1.81	SGR3091		**SCO4384/SCO4385/SCO4386**
SCO4428	hypothetical protein	gatcacgccg	5.35	-173	SLI4666	0.55	-		-
SCO4516	hypothetical protein	aattgcgccg	4.73	-184	SLI4797	0.374	-		**SCO4515**/SCO4514/SCO4513/**SCO4512/SCO4511**
SCO4671	LysR family transcriptional regulator	aggctggtac	4.7	-47	SLI4944	1.734	SGR1131		-
SCO4761	co-chaperonin GroES	aaccccgccg	3.31	-197	SLI5031	0.401	SGR2770	*groES*	-
SCO4762	chaperonin GroEL	ttgccgtata	4.4	-44	SLI5032	0.44	SGR2769	*groEL1*	-
SCO4768	two-component regulator	aatctagccg	5.52	-292	SLI5039	0.586	SGR2759	*bldM*	-

Additional file 5 – Putative *S. coelicolor* AdpA-binding sites upstream from the *S. lividans* AdpA-dependent genes[a].

gene[b]	function[c]	cis-element[c]	score[c]	position[c]	gene[d]	Fc[e]	gene[b]	gene name[f]	Co-transcript gene[g]
SCO4880	transferase	ggaccggcct	3.36	-138	SLI5154	0.597	SGR2660		SCO4881/**SCO4882**
SCO4951	aldoketoreductase	tgtcccgttt	5.58	-120	SLI5223	0.44	SGR7003		-
SCO4952	TetR family transcriptional regulator	tgtcccgttt	5.58	-35	SLI5224	0.301	SGR7004		**SCO4953**
SCO4974	deaminase	tattcggccc	5.63	-121	SLI5246	1.826	-		-
SCO5012	hypothetical protein	cgaccagcca	3.66	-72	SLI5288	0.466	SGR2522		**SCO5013/SCO5014 /SCO5015**
SCO5029	hypothetical protein	ttacccgccg	5.13	-377	SLI5306	0.566	SGR2504		-
SCO5050	nucleotide-sugar dehydrogenase	aaaccggcct	6.13	-37	SLI5327	1.852	-		**SCO5051/SCO5052 /SCO5053/SCO505 4**
SCO5101	hypothetical protein	cggcgggaac	6.11	-28	SLI5379	0.584	SGR2456		-
SCO5123	small membrane protein	atattggcca	6.23	-177	SLI5404	0.343	SGR2406		-
SCO5240*	hypothetical protein	tgtcccgatc	5.99	-170	SLI5531	2.246	SGR2274	*wblE*	-
SCO5249	nucleotide-binding protein	gtttcggaca	6.31	-187	SLI5540	0.551	SGR2264		-
SCO5390	alkanal monooxygenase	gaatcggccg	5.52	-119	SLI5659	0.608	SGR2147		-
SCO5512	acetolactate synthase 1 catalytic subunit	aatctcgcaa	5.09	-39	SLI5786	1.672	SGR2000	*ilvB*	**SCO5513**
SCO5555*	hypothetical protein	gtttcggcca	7.86	-241	SLI5832	2.828	SGR1927		-
SCO5556	histone-like DNA binding protein	ttgcggtata	3.76	-75	SLI5833	2.388	SGR1926	*hupS*	-
SCO5650	hypothetical protein	tgtcctttac	3.18	-14	SLI5907	0.578	SGR331		-
SCO5741	hypothetical protein	ggtccggatg	3.64	-90	SLI6001	2.502	SGR1779		-
SCO5742	hypothetical protein	ggactggaat	3.69	-32	SLI6002	2.094	SGR1778		-
SCO5751	hypothetical protein	tggcgtaaag	4.81	-97	SLI6012	0.613	SGR1770		-
SCO5790	hypothetical protein	gggcgaaatc	3.94	-35	SLI6052	1.704	-		SCO5789
SCO5862*	two-component regulator CutR	tggccgaaaa	7.69	-99	SLI6134	1.927	SGR1670	*cutR*	SCO5863
SCO6000	hypothetical protein	cggctggttc	5.53	-155	SLI6389	0.56	-		**SCO6001**
SCO6004	ATP/GTP binding protein	cggccgcatt	5.21	-292	SLI6392	0.603	**SGR1503**		-
SCO6009	solute-binding protein	cttccagcca	6.53	-52	SLI6398	1.736	SGR1498		-
SCO6073	cyclase	gggctgattc	4.27	-75	SLI6466	0.466	SGR6839	*geoA*	-
SCO6096*	lipoprotein	catcgcgcca	5.56	-147	SLI6490	0.557	SGR1397		**SCO6095**
SCO6099	adenylylsulfate kinase	tggccggtcc	5.72	-61	SLI6494	0.544	SGR1394	*cysC*	SCO6098/SCO6097
SCO6102	nitrite/sulfite reductase	cggccgaaaa	5.85	-366	SLI6496	0.532	SGR1391		SCO6101/SCO6100
SCO6174	hypothetical protein	ggaccggaac	4.01	-10	SLI6567	0.538	SGR1351		**SCO6175**
SCO6176	hypothetical protein	gaatcggcct	5.0	-79	SLI6569	0.459	-		-
SCO6197	hypothetical protein	aattcagcca	7.85	-117	**SLI6586**	0.147	-		-
SCO6198	hypothetical protein	aattcagcca	7.85	-470	**SLI6587**	0.618	SGR4455		-

Additional file 5 – Putative *S. coelicolor* AdpA-binding sites upstream from the *S. lividans* AdpA-dependent genes[a].

gene[b]	function[c]	cis-element[c]	score[c]	position[c]	gene[d]	Fc[e]	gene[b]	gene name[f]	Co-transcript gene[g]
SCO6376	hypothetical protein	cggccggacc	4.69	-59	**-**	0.603	-		-
SCO6384	integral membrane lysyl-tRNA synthetase	tggcaggatg	4.82	-122	**-**	0.59	-		-
SCO6482	hypothetical protein	tggcccgaac	7.23	-37	SLI6829	0.538	SGR1161		-
SCO6509	hydrophobic protein	tttcccgatt	4.04	-36	SLI6857	0.398	-		-
SCO6510	hypothetical protein	ttgctgtttt	3.44	-69	SLI6858	0.406	SGR1146		-
**SCO6685***	two-component system response regulator	gttcgggcca	7.23	-31	**SLI7029**	0.624	**SGR2393**	*ramR. amfR*	-
SCO6691	phospholipase C	cggccggttg	4.91	-41	SLI7035	0.414	SGR887		-
SCO6799	L-threonine 3-dehydrogenase	gggccgcttc	3.47	-55	SLI7149	1.638	SGR1444	*tdh*	**SCO6800/SCO6801/SCO6802/SCO6803/SCO6804**
SCO6808	transcription regulator ArsR	tggccggact	6.93	-77	SLI1107	0.268	-		-
SCO6820	oxidoreductase	tgtcggatac	4.86	-287	SLI1095	0.338	-		-
SCO6831	hypothetical protein	gttccggcaa	6.24	-241	SLI1083	0.602	SGR6080		-
SCO6903	hypothetical protein	cgtcccgatc	4.15	-43	SLI0959	0.617	-		**SCO6904**
SCO6926	hypothetical protein	tggcttatac	5.26	-394	SLI0925	0.6	-		-
SCO6979	solute-binding lipoprotein	tatccggtcc	4.15	-103	SLI7181	0.611	-		**SCO6980/SCO6981/SCO6982**
SCO7070	hypothetical protein	tgacgcgatc	4.69	-24	SLI7275	1.649	SGR1238		-
SCO7221	polyketide synthase	tggccaagtc	3.44	-169	SLI7437	1.692	-		-
SCO7251	hypothetical protein	tggcgcattc	5.36	-280	SLI7467	0.574	SGR6841		-
SCO7410	binding-protein dependent transport protein	tggcggcatg	4.67	-114	SLI7629	1.707	-		SCO7409
SCO7449	hypothetical protein	cggcgtgttc	4.54	-131	SLI7668	1.634	-		-
SCO7477	hypothetical protein	caagaggcca	3.43	-164	SLI7697	1.657	SGR1156		-
SCO7549	hypothetical protein	gttcccgcag	3.76	-49	SLI7771	0.447	-		-
SCO7550	hydrolase	gaaccggtca	5.88	-117	SLI7772	0.334	-	*glpQ3*	-
SCO7631*	hypothetical protein	tggcggtaac	5.97	-123	SLI7859	0.355	-		-
SCO7657*	hypothetical protein	gatccggcca	8.9	-139	**SLI7885**	0.033	**SGR3840**	*hyaS*	SCO7658
SCO7659*	oxidoreductase	gatcgagccg	4.98	-25	SLI7886	0.511	-		-
SCO7697	hydrolase	tggccgtcac	3.1	-36	SLI7928	0.557	-		-
SCO7714	acetyltransferase	gaagtcgcca	3.98	-110	SLI7945	0.516	-		-
SCO7774	hypothetical protein	cggcggaaag	4.04	-540	SLI8023	0.589	-		**SCO7775**

a. Orthologs of *S. lividans* AdpA-dependent genes (listed in Additional file 2) were analysed *in silico* using the *S. coelicolor* genome database (version 1.2.3.0 of PREDetector software [39]). AdpA-binding sites upstream from *S. coelicolor* genes were identified and are presented in this Additional file with information about their *S. lividans* and *griseus* orthologs. Table 3 presents a selected subset of this complete compilation mainly genes whose orthologs are probably direct *S. griseus* AdpA-dependent genes (gene underlined) [12-15, 22].

b. Gene names are from the StrepDB database [7]. Genes in bold were identified as direct AdpA-targets. Genes underlined were described as AdpA-dependent, putative AdpA-binding sites have been found *in silico* in the promoter of genes indicated by a star [12-15, 22].

c. Function, cis-element sequence, score and position were searched in the "upstream region" of *S. coelicolor* genome as given by PREDetector software (version 1.2.3.0)[39]. When more than one putative AdpA-binding site was detected, only the one with the highest score was shown here. # Putative sites were searched in the "coding region" and were located in the following coding sequences: SCO1033 (at position 250th/285nt total length gene) and SCO3122 (1447th/1449nt).

d. Gene names were identified by searching *S. coelicolor* orthologs in the StrepDB database [7]. Genes in bold were identified as direct *S. lividans* AdpA-targets in our studies. No *S. lividans* orthologs were found for the following *S. coelicolor* genes: SCO3495, SCO6376, SCO6384, SCO7449 that had Fc values closed to our microarrays cut-off. By blast we detected putative *S. lividans* genes orthologs to SCO1379, SCO2212 and SCO2921 (97-100% nucleotide homology). As Fc-values are significant for SCO1379 and SCO2921, it is likely that *S. lividans* genome carried orthologs of these genes that are *S. lividans* AdpA-dependent.

e. Gene expression fold change (Fc) in *S. lividans adpA* mutant compared to the wild-type strain 1326.

f. Other *Streptomyces* gene names from StrepDB database [7].

g. *S. coelicolor* co-transcript genes given by PREDetector software [39]. The genes in bold had no significant gene expression changes in *S. lividans adpA* mutant.
